# Protease‐Triggered Chromogenic Release From Peptide‐Modified Calcium Carbonate Microspheres Enables Colorimetric Detection of *Porphyromonas gingivalis* Activity

**DOI:** 10.1002/advs.77102

**Published:** 2026-08-18

**Authors:** Yuki Hiruta, Zhicheng Jin, Mari Adachi, Lubna Amer, Natalia O. Tjokro, Diego F. Trujillo, Jan Potempa, Anthony J. O'Donoghue, Casey Chen, Jesse V. Jokerst

**Affiliations:** ^1^ Aiiso Yufeng Li Family Department of Chemical and Nano Engineering University of California San Diego California USA; ^2^ Department of Applied Chemistry Faculty of Science and Technology Keio University Yokohama Japan; ^3^ School of Biological Sciences Molecular and Cell Biology University of California San Diego California USA; ^4^ Program in Materials Science and Engineering University of California San Diego California USA; ^5^ Herman Ostrow School of Dentistry University of Southern California Los Angeles USA; ^6^ Skaggs School of Pharmacy and Pharmaceutical Sciences University of California San Diego California USA; ^7^ Department of Microbiology Faculty of Biochemistry Biophysics and Biotechnology Jagiellonian University Krakow Poland; ^8^ Department of Oral Immunology and Infectious Diseases School of Dentistry University of Louisville Louisville USA

**Keywords:** biosensors, calcium carbonate, chromogenic release‐based assay, *P. gingivalis*, peptides

## Abstract

*Porphyromonas (P.) gingivalis* is a Gram‐negative anaerobic bacterium with exceptionally high pathogenicity among periodontal pathogens. It is associated not only with periodontal disease but also with various systemic diseases, including Alzheimer's disease, rheumatoid arthritis, and atherosclerosis. Therefore, early detection of this bacterium is crucial for enabling subsequent treatment and prevention of such conditions. Herein, we report a chromogenic release‐based assay system utilizing calcium carbonate microspheres and a dye‐labeled peptide that is selectively cleaved by an Arg‐specific protease called gingipain (RgpB) that is secreted by *P. gingivalis*. By utilizing the reactive anhydride functional groups of poly(isobutylene‐*alt*‐maleic anhydride), we simultaneously achieved the conjugation of the dye‐labeled peptide and its immobilization onto calcium carbonate microspheres. Through optimization of the peptide sequence and the surface modification density on calcium carbonate microspheres, the assay of RgpB detection with the absorbance of supernatant exhibited a low limit of detection of 0.25 nM, as well as high storage stability and selectivity for *P. gingivalis*. The measurements obtained with this assay system for clinical gingival crevicular fluid samples showed a high correlation (Pearson's *r* = 0.79) with qPCR results. This assay system offers an on‐site, visual means to monitor *P. gingivalis* activity without requiring specialized instrumentation.

## Graphical abstract

We report a chromogenic release‐based assay system with protease‐cleavage peptide‐modified calcium carbonate microspheres for colorimetric detection of *Porphyromonas (P.) gingivalis* activity. In response to Arg‐specific gingipain secreted by *P. gingivalis*, the supernatant becomes colored, enabling colorimetric analysis and providing the monitoring of its activity without requiring specialized instrumentation.

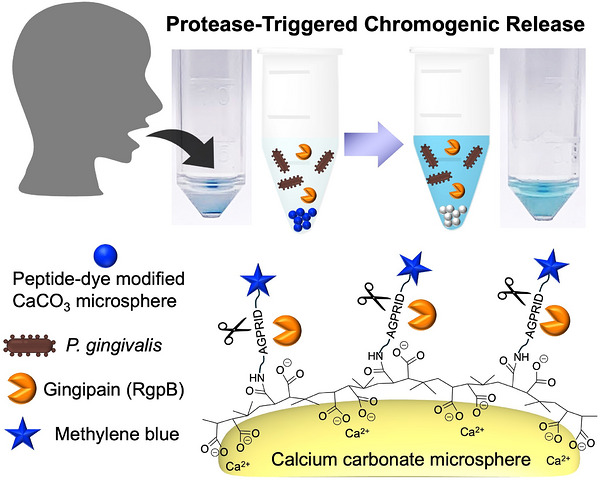

## 1 Introduction

Periodontal disease is a chronic inflammatory disorder that affects the tissues supporting the teeth and is estimated to affect approximately 19% of the global adult population, representing more than 1 billion cases worldwide [1]. Gingival inflammation is induced by the dysbiotic accumulation of multispecies bacterial biofilms at the gingival margin, which elicits destructive immune‐inflammatory responses [2]. Among the bacteria that cause periodontal disease, three species, *Porphyromonas (P.) gingivalis*, *Tanerella forsythia*, and *Treponema denticola*, are classified as in the “red complex” and are strongly associated with severe progression of periodontal disease [3]. Notably, *P. gingivalis*, a Gram‐negative anaerobic bacterium, has been recognized as a key periodontal pathogen [4] and has also been implicated in various systemic diseases beyond periodontitis, such as Alzheimer's disease [5], rheumatoid arthritis [6], and cardiovascular diseases [7].

These studies imply that the early detection and control of *P. gingivalis* may reduce the risk of not only dental diseases, but also systemic diseases such as Alzheimer's disease. Therefore, it is important to detect, and treat such conditions at an early stage even before the onset of symptoms. Although PCR is commonly used to monitor the presence and amount of *P. gingivalis*, it requires specialized operators and involves time and cost for sample transportation, making it unsuitable for routine clinical monitoring of *P. gingivalis*. To monitor the activity of *P. gingivalis*, plasmonic colorimetric nanosensors [8, 9, 10], and fluorescence sensors [11] have been developed, which utilize peptide cleavage and proteolytic degradation of the protein by gingipains secreted by *P. gingivalis*. Plasmonic colorimetric nanosensors are often sensitive to matrix effects, as complex biological fluids can significantly influence nanoparticle aggregation and dispersion behaviors. Consequently, the reliable application of these sensors to biological fluids, including blood, urine, and saliva, remains challenging [12]. Indeed, electrolytes and proteins in saliva have been reported to interfere with nanoparticle responses, resulting in suppressed target‐induced signals as well as nonspecific or false signals [13]. Similarly, fluorescence‐based sensors are susceptible to matrix‐derived optical interference, including autofluorescence and inner filter effects arising from endogenous components in biological samples [14]. Such matrix effects have been reported to influence fluorescence measurements in real saliva samples [15].

Colorimetric analysis enables the rapid acquisition of analytical results through color changes [16]. In recent years, sensitive and portable sensors have been developed [17]. In addition to semi‐quantitative analysis by the naked eye, quantitative analysis using smartphone cameras has also become possible [18], making this method well suited for point‐of‐care testing. Among them, chromogenic release‐based assays use insoluble particles or hydrogels on which a colored dye is immobilized. Enzymatic cleavage releases dye‐labeled fragments into solution, allowing straightforward quantification of enzyme activity by measuring the color intensity of the supernatant [19, 20, 21]. Chromogenic polysaccharide hydrogel substrates in conjugation with a 96‐well filter plate form a high‐throughput enzyme activity assay system [19]. One such commercially available product (Phadebas tablets) is used for the accurate determination of α‐amylase activity with a chromogenic release‐based assay [20]. In a recent study, Phadebas tablets were applied to face masks to detect α‐amylase in deposited saliva, enabling noninvasive, colorimetric mapping of respiratory aerosols on face coverings [22], which implies practical tolerance of chromogenic release‐based assays to sample matrix effects in biological samples.

Here, we report protease‐responsive calcium carbonate microspheres that release dye‐labeled peptide fragments into the solution upon enzymatic cleavage, demonstrating their potential as a simple and robust colorimetric platform for monitoring enzymatic activity from *P. gingivalis* without complex reagent preparation or handling. CaCO_3_ is commonly used as an abrasive and cleaning agent in oral care products such as toothpaste. Thus, this strategy could potentially be adapted for such applications, providing a simple, on‐site, and visual means to monitor *P. gingivalis* activity without requiring specialized instrumentation.

## 2 Results and Discussion

*P. gingivalis* secretes a family of cysteine proteases called gingipains, which play a crucial role in its extracellular proteolytic activity [23]. They account for approximately 85% of the total proteolytic activity of *P. gingivalis* [24]. The gingipain family consists of Arg‐specific gingipains (RgpA and RgpB), which cleave peptide bonds at the C‐terminal side of arginine residues, and Lys‐specific gingipain (Kgp), which cleaves the C‐terminal side of lysine. Among the arginine‐gingipains, RgpA contains both proteolytic and adhesion domains, whereas RgpB is composed solely of a catalytic domain [25], enabling a more direct and reproducible evaluation of proteolytic activity. Therefore, RgpB was selected as the primary target for monitoring *P. gingivalis* activity.

**FIGURE 1:**
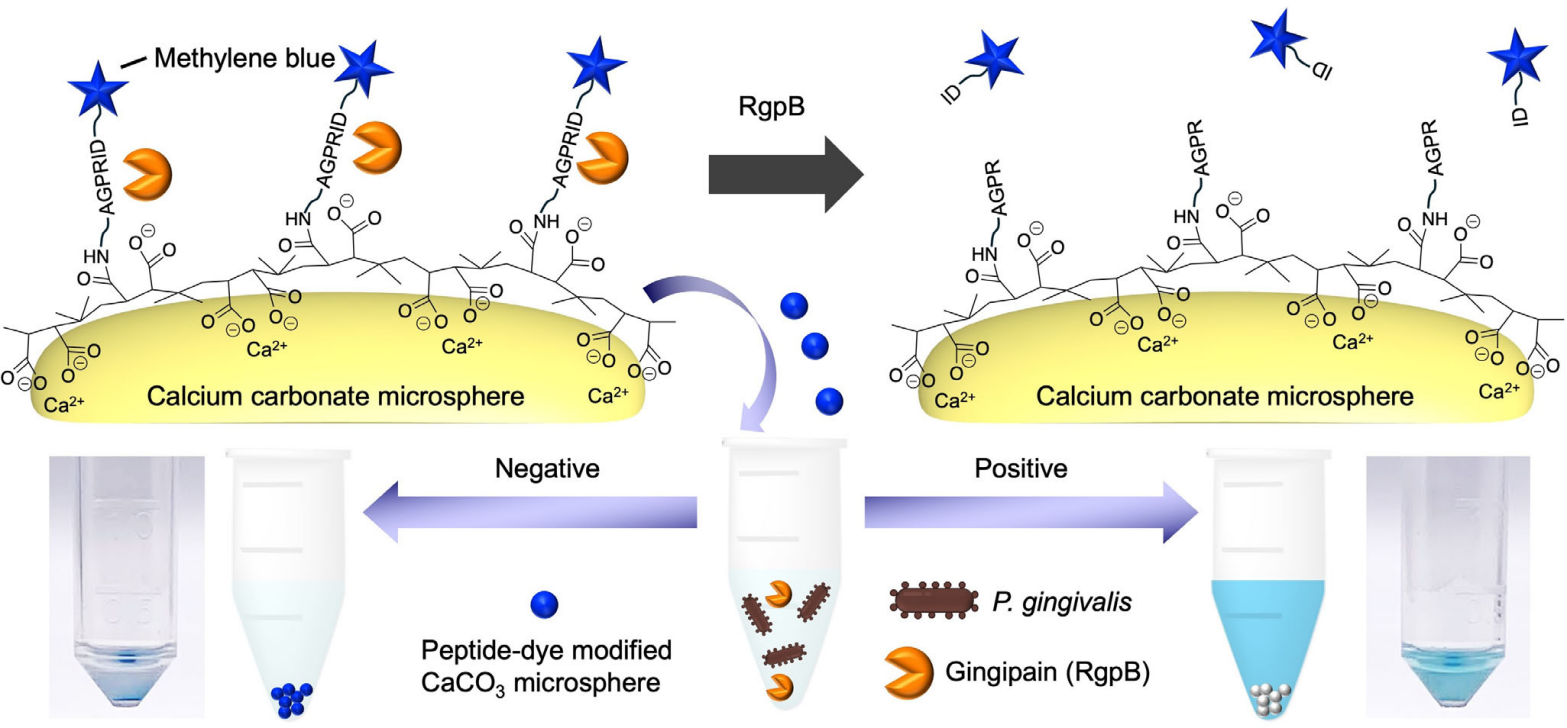
Schematic representation of PIMA‐peptide‐MB modified calcium carbonate microsphere for chromogenic release‐based colorimetric detection of gingipain (RgpB). In aqueous solution, the microspheres sediment, resulting in a colored precipitate and a transparent supernatant in negative samples. In contrast, in positive samples containing RgpB secreted by *P. gingivalis*, MB‐labeled peptide fragments are released into the supernatant, producing a blue‐colored supernatant. This assay allows detection of RgpB, providing a colorimetric readout of *P. gingivalis* activity, without complex reagent preparation and handling.

The design of RgpB‐responsive CaCO_3_ microspheres for a chromogenic release‐based assay for *P. gingivalis* is illustrated in Figure 1. CaCO_3_ was selected as the microsphere material because it is inexpensive, biocompatible, and readily scalable to large‐scale production. CaCO_3_ microspheres were modified with a poly(isobutylene‐*alt*‐maleic anhydride) (PIMA)‐based polymer in which some of the maleic anhydride units were conjugated with the ε‐amino group of an N‐terminal lysine residue in RgpB‐cleavable peptides labeled with a water‐soluble dye, methylene blue (MB). The remaining maleic anhydride units were hydrolyzed to carboxyl groups, resulting in the poly(isobutylene‐*alt*‐maleic acid) backbone. The carboxyl groups interact electrostatically with Ca^2+^, providing stable surface adsorption. In the absence of RgpB, the microspheres remain as a colored precipitate, whereas in the presence of RgpB, the dye‐labeled peptide fragments are released into the supernatant, producing a visible color change in the sample solution. This allows simple colorimetric detection of RgpB activity without specialized instrumentation. In addition, MB is monitored through absorbance at a relatively long wavelength (>650 nm), where interference from endogenous chromophores present in biological samples is generally reduced.

RgpB is an endopeptidase that can cleave peptides on the C‐terminal side of arginine (R). While several sequences have been reported as substrates of RgpB [8, 11], the AGPR/ID is one of the peptide sequences cleaved by RgpB with high efficiency [26]. The C‐terminus of this peptide sequence was designed to introduce MB via maleimide‐thiol coupling, with either a di‐glycine (GG) or di‐glycine‐di‐aspartic acid (GGDD) spacer followed by a cysteine residue. The GG spacer was introduced to provide flexibility, preventing steric hindrance of RgpB recognition when MB is directly attached. The DD sequence was added to introduce two negative charges. Previously, it was reported that when MB‐modified peptide ligands, cleavable by a target protease, were immobilized on quantum dots (QDs) functionalized with carboxylic acid‐containing ligands, the presence of aspartic acid residues reduced re‐adsorption of the MB‐labeled peptide fragments after protease cleavage, leading to enhanced fluorescence activation [27]. In this study, the RgpB‐responsive CaCO_3_ microsphere surface contains both positively charged Ca^2+^ ions (as bare CaCO_3_ surfaces are known to exhibit a weakly positive surface charge under near‐neutral aqueous conditions [28]; however, the zeta potential of the microspheres used in this study could not be measured because their size was too large for reliable zeta‐potential analysis) and negatively charged carboxyl groups derived from maleic acid residue. We hypothesized that the charge of the cleaved fragments would significantly affect their release into solution after RgpB‐mediated cleavage and thus compared their release behaviors. The N‐terminus of the peptide conjugated to PIMA was designed to include an acetylated lysine residue linked via a polyproline (PPP) linker. Polyproline acts as a rigid rod [29] and is a spacer that reduces steric hindrance on the nanoparticle surface to facilitate proteolytic activation [30]. In this study, the polyproline linker was introduced to increase the distance between the PIMA backbone and the cleavage peptide sequence, thereby preventing interference with RgpB recognition. Based on the concepts described above, in this study, we have synthesized KPPPAGPRIDGGC (gingipain‐cleavable peptide: GcP) and KPPPAGPRIDGGDDC (GcPDD) to directly assess the effect of fragment charge on release behavior after RgpB‐mediated cleavage (Table 1).

**TABLE 1:** The information of peptide and peptide‐MB conjugate.

Peptide name	Peptide sequence^a^	M. W. [g/mol]	Net charge^b^
GcP	KPPPAGPR/IDGGC	1304.7	+1
GcPDD	KPPPAGPR/IDGGDDC	1534.7	−1
GcP‐MB	KPPPAGPR/IDGGC‐MB	1782.9	+2
GcPDD‐MB	KPPPAGPR/IDGGDDC‐MB	2012.9	0

^a^ All peptides and peptide‐dye conjugates contain an acetylated N‐terminus and an amidated C‐terminus

^b^ The electrophoretic property of the peptide at pH 8.0.

GcP and GcPDD were synthesized by standard Fmoc‐based solid‐phase peptide synthesis and purified using reversed‐phase high‐performance liquid chromatography (HPLC). The syntheses of these peptides were confirmed by electrospray ionization mass spectrometry (ESI‐MS), and the chromatograms indicated that both peptides had high purity (Table 1; Figure S1). When GcP was treated with RgpB, the treated sample showed two peaks at 4 min (Fr. 1 in Figure S2a) and 7 min (Fr. 2 in Figure S2a) in the chromatogram, whereas a single peak was observed at 14 min for the untreated sample (Figure S2a). Furthermore, ESI‐MS analysis identified fractions as [IDGGC+H]^+^ and [KPPPAGPR+2H]^2+^ (Figure S2b,c), respectively, indicating that cleavage occurred at the C‐terminal side of arginine (R) as designed. For GcPDD as well, similar results to those of GcP were obtained (Figure S3), except that due to the influence of the di‐aspartic acid residue, IDGGDDC (Fr. 1 in Figure S3a) was not detected in the positive mode, but was observed in the negative mode as [IDGGDDC–H]^−^ (Figure S3b).

The RgpB‐responsive peptide‐MB conjugates, GcP‐MB and GcPDD‐MB, were also confirmed by ESI‐MS, and the chromatograms showed a dominant single peak, indicating high conjugation purity (Table 1; Figure S4). In the ESI‐MS spectra of GcP and GcPDD, signals of doubly charged ions were predominantly observed, whereas when MB was conjugated to the peptide, the signals were mainly observed as triply charged ions, likely due to the influence of the positive charge of MB. After treatment of GcP‐MB and GcPDD‐MB with RgpB, the cleaved peptide‐MB fragments were detected at 650 nm and subsequently collected for identification by ESI‐MS (Figures S5 and S6). The ESI‐MS spectra confirmed that both peptide‐MB conjugates were cleaved at the same site as the corresponding peptides, indicating that the conjugation of MB did not affect the recognition by RgpB.

**FIGURE 2:**
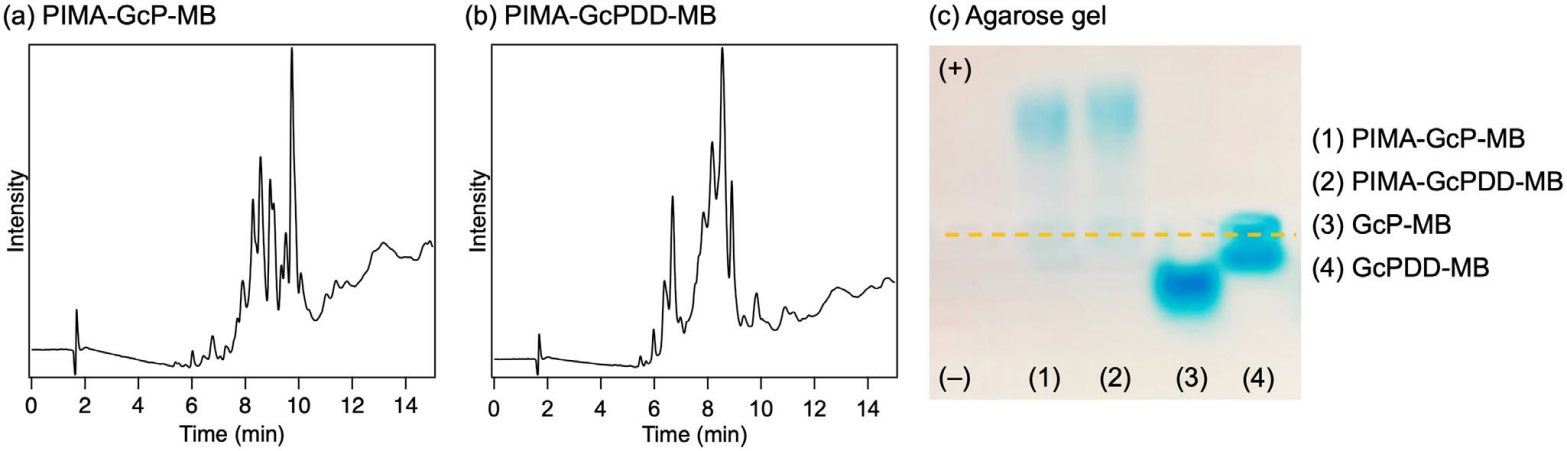
Confirmation of the conjugation between PIMA and peptide‐MB. (a) The chromatograms of (a) PIMA‐GcP‐MB and (b) PIMA‐GcPDD‐MB. Analytical conditions: 0.05% TFA water/acetonitrile starting with 90/10 (v/v) followed by gradient elution to 60/40 (v/v) for 15 min, 1.0 mL/min, 20 µL injection of 6 µM PIMA‐peptide‐MB conjugates, detected 650 nm. (c) Agarose gel electrophoresis acquired from peptide‐MB conjugates and PIMA‐peptide‐MB conjugates. The yellow dashed line indicates the sample loading position.

PIMA has highly reactive anhydride functional groups (∼40) that could easily be conjugated to multifunctional groups with the mild reaction condition [31]. Previously, cleavable peptide sequence and FRET pair conjugates have been introduced into PIMA for applications to monitoring enzyme activity [32, 33]. In addition, ring‐opening hydrolysis of maleic anhydride and the introduction of functional substituents have been utilized to modify the surface properties of particles [34, 35]. The peptide‐MB conjugates were conjugated to PIMA through the reaction between the ε‐amino group of an N‐terminal lysine residue and the maleic anhydride groups of PIMA. Subsequently, the unreacted maleic anhydride groups were hydrolyzed in NaOH aqueous solution to form maleic acid. The conjugation of the peptide‐MB conjugates to PIMA was confirmed by HPLC and agarose gel electrophoresis (Figure 2). The chromatogram showed multiple peaks and broad tailing, which can be attributed to the polydispersity of PIMA and the variation in the number of peptide‐MB conjugates attached to each PIMA chain. In agarose gel electrophoresis under buffer conditions (pH 8.3), GcP‐MB and GcPDD‐MB exhibited a net charge of +2 and 0, respectively (Table 1), and their electrophoretic migrations corresponded accordingly. In contrast, after conjugation with PIMA, the products electrophoretically migrated broadly toward the positive electrode, which can be explained by the presence of multiple negatively charged maleic acid groups generated through hydrolysis and the polydispersity of PIMA. These results indicate that the peptide‐MB conjugates were successfully conjugated to PIMA and the remaining maleic anhydride groups were hydrolyzed to maleic acid.

**FIGURE 3:**
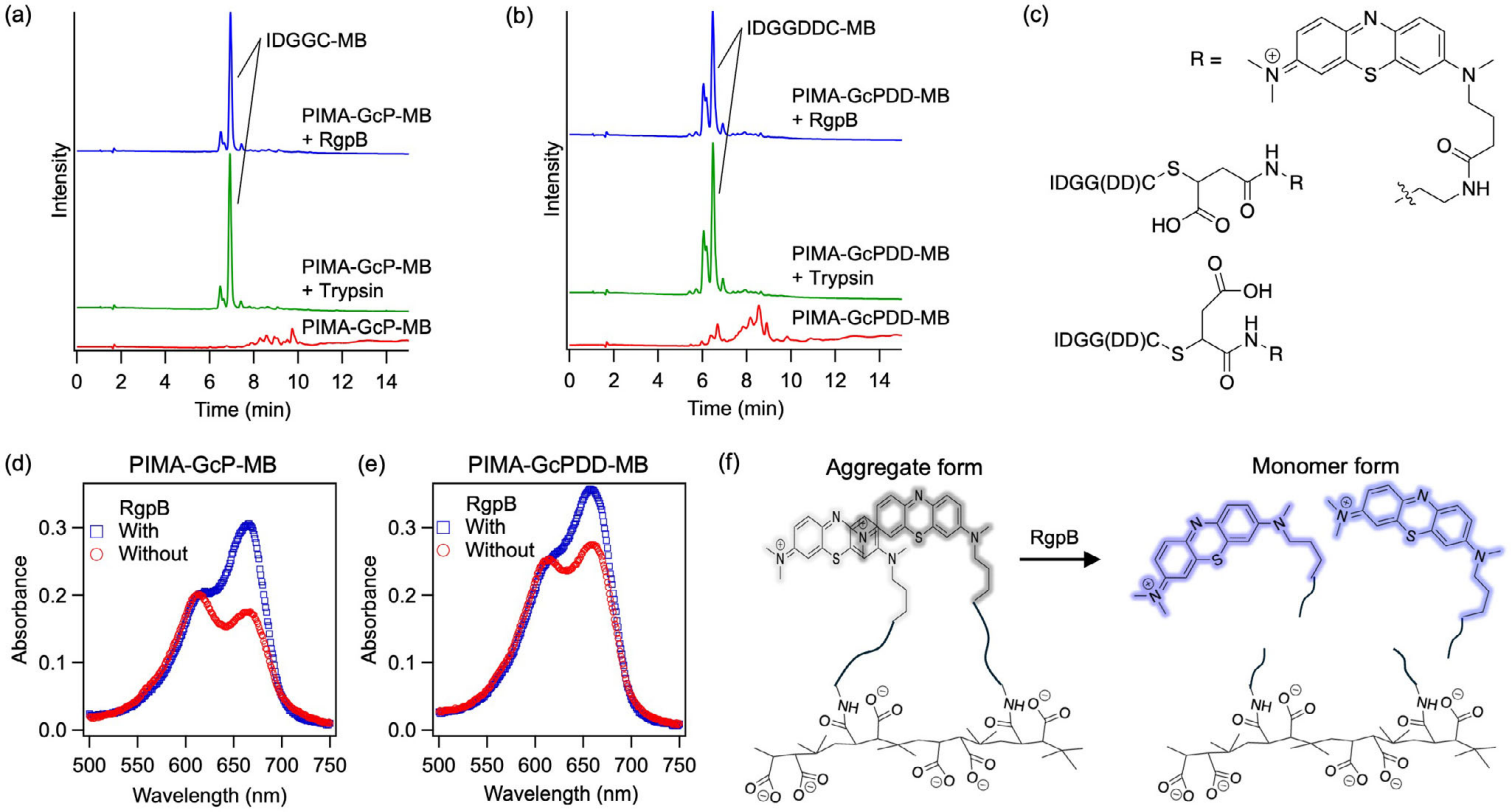
(a, b) Chromatograms of PIMA‐GcP(DD)‐MB (6 µM) in response to trypsin (2 µM) and RgpB (192 nM). Analytical conditions: 0.05% TFA water/acetonitrile starting with 90/10 (v/v) followed by gradient elution to 60/40 (v/v) for 15 min, 1.0 mL/min, 20 µL injection, detected 650 nm. (c) Chemical structure of main peak with trypsin or RgpB cleaved assumed by ESI‐MS analysis. (d, e) Absorbance spectra of PIMA‐GcP(DD)‐MB (6 µM) with(out) RgpB (192 nM). (f) Schematic representation of the transition from the aggregated MB to monomeric MB upon cleavage of the peptide linker by RgpB. All experiments were performed in Tris buffer (20 mM, pH 7.6, with 100 mM NaCl, 5 mM CaCl_2_, 1 mM DTT).

The protease reactivity of PIMA‐peptide‐MB conjugates was evaluated using trypsin and RgpB. Trypsin is a pancreatic digestive protease that cleaves the peptides at the C‐terminal side of an arginine residue and is not present in the oral cavity. Since PIMA‐peptide‐MB is a polymer possessing multiple negative charges derived from the maleic acid moieties, the interaction with proteases may be influenced by their size and net charge. Trypsin (23.4 kDa, isoelectric point pI 10.8) [36] and RgpB (48.8 kDa, pI 5.1) [37] differ substantially in both molecular weight and charge properties and were used to assess these effects. When PIMA‐GcP(DD)‐MB was treated with trypsin or RgpB, the multiple peaks in the chromatogram originating from the polydispersity of the PIMA backbone disappeared, and sharp single peaks were obtained (Figure 3a,b). The resulting peak fractions were analyzed by ESI‐MS, which showed molecular weights of 479.8 (PIMA‐GcP‐MB) and 595.3 (PIMA‐GcPDD‐MB), respectively (Figure S7). These values differed from those observed in the ESI‐MS spectra of GcP‐MB and GcPDD‐MB after RgpB treatment (471.1 and 586.2 in Figures S5b and S6b, respectively). These differences corresponded to the molecular weight of a water molecule, suggesting that the succinimidyl thioether moiety also underwent ring‐opening hydrolysis to provide two isomeric succinamic acid thioethers (Figure 3c) [38] during the hydrolysis of maleic anhydride residues in NaOH aqueous solution. As a result, the same site was cleaved when the peptide was conjugated to PIMA. Since PIMA‐peptide‐MBs were recognized and cleaved even by protease with markedly different molecular weights and pI values, it was demonstrated that conjugation to PIMA did not interfere with enzyme recognition.

The absorption spectra were measured to evaluate how the optical properties of MB change upon conjugation to PIMA and subsequent peptide cleavage by protease. MB exhibits a monomer absorption maximum around 665 nm in diluted aqueous solutions [39]. In contrast, at higher concentrations, MB is known to undergo concentration‐dependent dimerization, accompanied by the appearance of a blue‐shifted absorption band [40]. Even at concentrations where free MB remains monomeric, conjugation to PIMA led to the appearance of an additional dimer peak around 615 nm in PIMA‐GcP‐MB (Figure 3d). Because multiple MB molecules are attached to the PIMA backbone, they were in proximity and able to form dimers. A similar behavior was observed for PIMA‐GcPDD‐MB, although the proportion of the dimer peak was smaller (Figure 3e). This is likely because the two negative charges on DD induce electrostatic repulsion, which suppressed dimer formation. Upon cleavage of the peptide by RgpB and trypsin, the MB‐labeled peptide fragments were released from the PIMA backbone, and the dimer peak disappeared, and the monomer peak predominantly remained (Figure 3d,e; Figure S8).

After confirming the protease‐mediated cleavage behavior of PIMA‐GcP(DD)‐MB in solution, we next investigated its behavior on the surface of three types of porous CaCO_3_ microspheres (CAL‐LIGHT‐KT, CAL‐LIGHT‐SA, and PORECAL‐N), selected as representative inorganic substrates relevant to oral care applications. Their morphologies are shown in the scanning electron microscopy (SEM) images (Figure S9). We then evaluated them using a chromogenic release‐based assay. It has been reported that poly(maleic acid‐*alt*‐1‐octadecene) (PMAcO) can be robustly immobilized on CaCO_3_ surfaces simply by mixing, through electrostatic interactions with surface Ca^2+^, regardless of the crystal structure or morphology of CaCO_3_ [41, 42].

**FIGURE 4:**
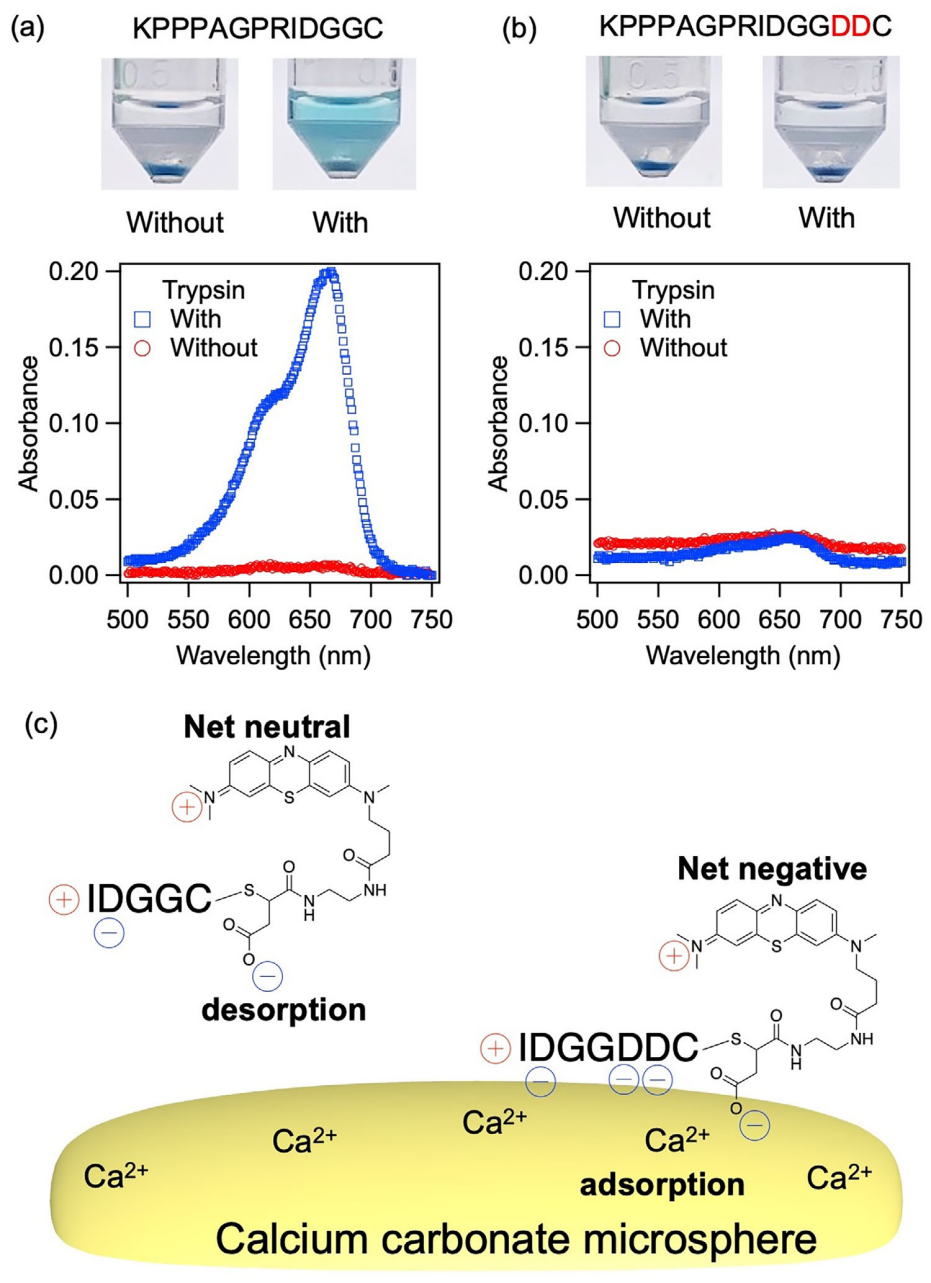
Colorimetric response of PIMA‐peptide‐dye modified CAL‐LIGHT‐KT microspheres (1.5 mg) to trypsin in Tris buffer (20 mM, pH 7.6, with 100 mM NaCl, 5 mM CaCl_2_, 1 mM DTT). (a, b) Images of sample solutions with microspheres after natural sedimentation post‐response to trypsin, and absorption spectra of the supernatants of (a) PIMA‐GcP‐MB modified CAL‐LIGHT‐KT, and (b) PIMA‐GcPDD‐MB modified CAL‐LIGHT‐KT. (c) Illustration of the differences in results between using PIMA‐GcP‐MB and PIMA‐GcPDD‐MB.

The poly(isobutylene‐*alt*‐maleic acid) backbone obtained after hydrolysis also possesses multiple negative charges, and thus the modification was performed simply by mixing in water in the same manner. First, the trypsin response of the PIMA‐GcP‐MB‐modified CaCO_3_ microspheres was evaluated. As shown in Figure 4a and Figure S10a,c, the supernatant remained completely colorless in the absence of trypsin, while only the CaCO_3_ microsphere precipitates appeared blue. This indicates that the surface modification of the CaCO_3_ microspheres through electrostatic interactions was sufficiently strong in the buffer solution. In contrast, in the presence of trypsin, blue coloration was observed in the supernatant. This is attributed to the peptide‐MB fragments generated by trypsin cleavage, which no longer adsorbed onto the CaCO_3_ microspheres and were released into the solution. An absorption peak derived from the MB monomer was observed. MALDI‐TOF‐MS analysis of the resulting supernatant revealed peaks derived from IDGGC‐MB (Figure S11), confirming that the peptide was selectively recognized and cleaved even on the surface of the CaCO_3_ microspheres.

On the other hand, when PIMA‐GcPDD‐MB was used, sufficient coloration of the supernatant could not be observed (Figure 4b; Figure S10b,d). PIMA‐GcPDD‐MB was recognized by trypsin and RgpB in solution, and IDGGDDC‐MB fragments were detected. These results suggest that the fragments remained adsorbed on the CaCO_3_ microsphere surface due to the influence of the di‐aspartic acid motif.

As described above, the PIMA‐peptide‐MB‐modified CaCO_3_ microsphere surface contains both positively charged Ca^2+^ ions and negatively charged carboxyl groups derived from maleic acid residue; in this case, electrostatic interactions with Ca^2+^ were dominant. Strong adsorption of aspartic‐acid‐rich peptides onto calcite has also been reported [43], and thus the IDGGDDC‐MB fragments were structurally expected to exhibit high affinity for the CaCO_3_ surface (Figure 4c). To confirm this hypothesis, the PIMA‐GcPDD‐MB‐modified CAL‐LIGHT‐KT samples shown in Figure 4b were washed to remove the supernatant, followed by the addition of hydrochloric acid to dissolve the calcium carbonate, and their absorption spectra were measured. In the trypsin‐untreated samples, absorption peaks originating from MB dimers were observed, whereas in the trypsin‐treated samples, peaks derived predominantly from the MB monomer were observed (Figure S12). These results indicate that although PIMA‐GcPDD‐MB underwent trypsin‐mediated cleavage on the CaCO_3_ surface, the released IDGGDDC‐MB fragment strongly adsorbed onto CaCO_3_.

**FIGURE 5:**
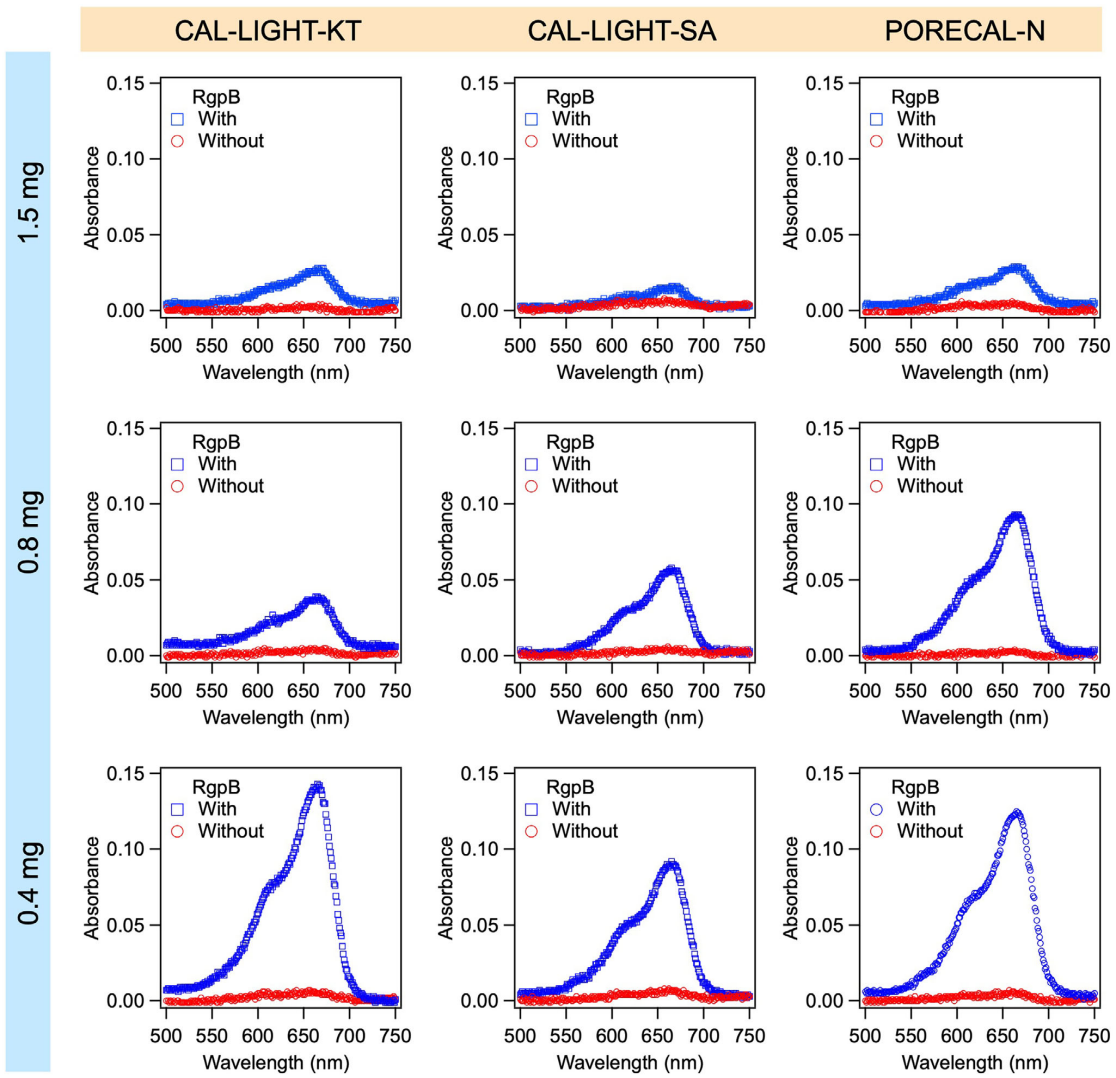
Colorimetric response of PIMA‐peptide‐dye‐modified CaCO_3_ microspheres to RgpB in Tris buffer (20 mM, pH 7.6, with 100 mM NaCl, 5 mM CaCl_2_, 1 mM DTT). Differences in the Absorption spectra of the supernatants depending on the type and amount of CaCO_3_ microspheres.

Based on the trypsin assay results, which confirmed that PIMA‐GcP‐MB was effective for the chromogenic release‐based assay on CaCO_3_ microspheres, subsequent experiments were performed using PIMA‐GcP‐MB to evaluate the RgpB assay. Although PIMA‐GcP‐MB showed similar responses to trypsin and RgpB in solution (Figure 3a), its response to RgpB was lower than that to trypsin once immobilized on CaCO_3_ (the conditions using 1.5 mg of CaCO_3_ microspheres in Figure 5 are identical to those in Figure 4a; Figure S10). Since the charge of the peptide strongly influenced its adsorption onto the CaCO_3_ surface, the surface charge of the proteins was also considered to significantly influence their adsorption, thus affecting their enzymatic responses. In contrast to trypsin (pI 10.8) [36], which is positively charged in the pH 7.6 buffer solution, RgpB (pI 5.1) [37] is negatively charged and might be strongly adsorbed to CaCO_3_ through electrostatic interactions with the positively charged Ca^2+^, leading to its reduced enzymatic response. To verify this hypothesis, residual RgpB activity remaining in the supernatant was measured after incubation with CaCO_3_ microspheres. Specifically, the cleavage of GcP by RgpB was compared with and without a prior incubation step with CaCO_3_ microspheres. As shown in Figure S13, GcP cleavage was markedly suppressed following microsphere incubation, indicating a substantial reduction in the amount of active RgpB remaining in the supernatant after treatment with CaCO_3_ microspheres.

To further investigate the influence of CaCO_3_ on the RgpB activity, we examined how the RgpB response changed when the amount of CaCO_3_ relative to PIMA‐GcP‐MB was reduced. The amount of PIMA‐GcP‐MB adsorbed onto the CaCO_3_ surface was calculated by measuring the absorbance of the supernatant obtained after the surface modification process (Figure S14 and Table S1). When the polymer‐to‐CaCO_3_ ratio was varied, three types of microspheres showed that increasing the amount of PIMA‐GcP‐MB per CaCO_3_ surface area, which corresponds to a decrease in the surface area of exposed CaCO_3_, resulted in a greater RgpB response (Figure 5). Although the zeta potential could not be directly determined in this work as mentioned above, previous studies have reported that modifying CaCO_3_ surfaces with anionic polymers gradually decreases the zeta potential, shifting it from positive to negative values as polymer coverage increases [44, 45]. A similar trend has been reported for alumina particles modified with poly(maleic acid), a structural analogue of the PIMA backbone used in this work [46]. Therefore, increasing the amount of PIMA‐GcP‐MB per unit surface area is expected to lower the surface potential. Based on the surface adsorption amount of PIMA‐GcP‐MB and the absorbance of peptide‐MB fragments released into the supernatant upon RgpB treatment, the recovery rates were calculated. Samples with a larger amount of CaCO_3_ exhibited only 6–12%, whereas reducing CaCO_3_ content increased the recovery with three types of microspheres. Under the condition with the lowest CaCO_3_ amount, the recovery rates reached 79–84% (Table S2). Although the Ca^2+^ surface sites are not fully occupied, the higher surface density of PIMA‐GcP‐MB likely disrupts continuous Ca^2+^ domains, thereby suppressing multivalent RgpB adsorption and preserving enzymatic activity. Notably, the surface density of isobutylene‐*alt*‐maleic acid units (∼0.50 molecules/nm^2^) is still significantly lower than the intrinsic surface Ca^2+^ site density of CaCO_3_ (approximately 5–6 sites/nm^2^) [47], indicating that the observed effect does not arise from complete coverage of Ca^2+^ sites but rather from disruption of continuous exposed Ca^2+^ domains at the nanoscale. MALDI‐TOF‐MS analysis of the resulting supernatant revealed peaks corresponding to IDGGC‐MB (Figure S15), confirming that RgpB cleaved the same peptide sequence as trypsin.

These results suggest that reducing the exposed CaCO_3_ surface suppressed the adsorption of RgpB, thereby maintaining its enzymatic activity. Among the nine conditions tested, comparing three types of microspheres and three polymer‐to‐CaCO_3_ ratios, the highest absorbance was observed for 0.4 mg of CAL‐LIGHT‐KT condition, which was selected for subsequent RgpB sensing applications.

**FIGURE 6:**
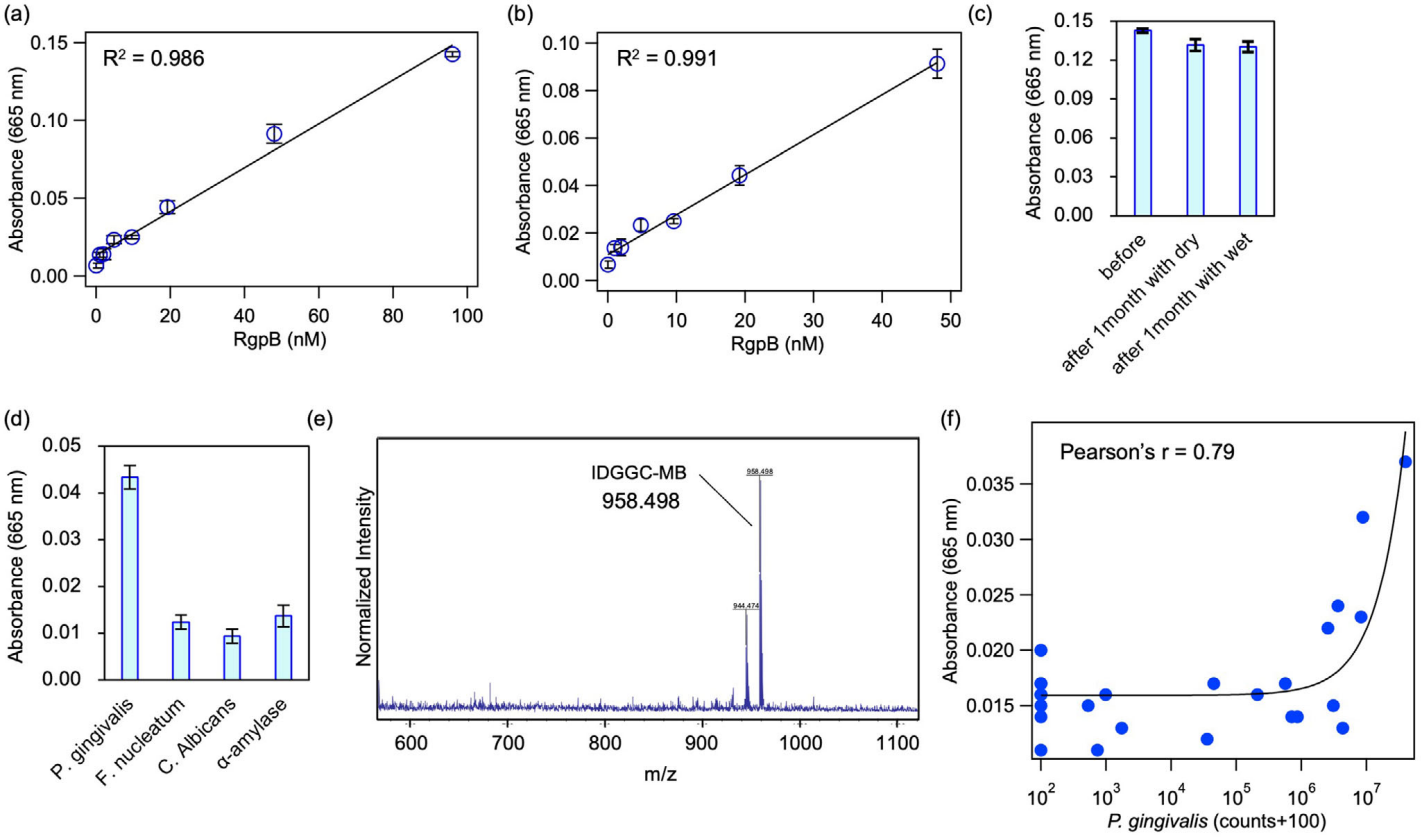
Colorimetric response of PIMA‐GcP‐MB modified CaCO_3_ microspheres to RgpB. Absorbance of the supernatants depending on [RgpB]: (a) concentration range 0–96 nM; (b) enlarged view of the 0–48 nM range. (c) Storage stability of PIMA‐GcP‐MB modified CaCO_3_ microspheres. Comparison of the absorbance of the supernatants after treatment with 96 nM RgpB following storage by each method. (d) Evaluation of the selectivity to RgpB in bacterial cultures and control microorganisms and α‐amylase. Comparison of the absorbance of the supernatants after treatment with these samples. The error bars represent the standard deviation of three independent replicates (*n* = 3). (e) MALDI‐MS spectra of the supernatants after treatment with *P. gingivalis*. (f) Correlation between the absorbance variation of the supernatant and the number of *P. gingivalis* cells detected by qPCR. To include samples with zero counts, all values were shifted by adding 100, and the x‐axis is shown as *P. gingivalis* (counts + 100).

The absorption spectra of the supernatant at different RgpB concentrations are shown in Figure S16. As the RgpB concentration increased, the absorbance became higher due to the increased amount of peptide–MB fragments released into the supernatant, resulting in the response curve shown in Figure 6a,b. While high linearity (*R* ^2^ = 0.991) is maintained in the 0–48 nM range, plotting the data up to 96 nM still exhibits a reasonable linear correlation (*R* ^2^ = 0.986), although with a slope approximately 17% lower than that observed in the 0–48 nM range. Under the conditions of this assay, an MB recovery rate of 79% was reached at 96 nM RgpB (Table S2), indicating that almost all sterically accessible peptide‐MB conjugates on the interface were successfully cleaved and released by RgpB. Therefore, the near‐complete consumption of available peptide‐MB substrates is considered to have caused the lower linearity observed over the wider 0–96 nM range. The limit of detection (LOD) of RgpB was determined from the calibration curve obtained over the linear range (*R* ^2^ = 0.991) of 0–48 nM, using the value of mean_blank_ + 3σ, and was found to be 0.25 nM. It has been reported that surface‐tethered protease sensors often suffer from steric hindrance, which can be mitigated by integrating homogeneous reactions to enhance sensitivity [48, 49]. In our system, although the peptides are immobilized on the microsphere surface, the LOD (0.25 nM) remains lower than that of a fluorescence nanosensor (1.1 nM) [11] and is comparable to that of a plasmonic colorimetric nanosensor (0.1 nM) [8], which also uses peptide cleavage. These results suggest that our structural design, incorporating a flexible PIMA polymer and a peptide spacer, successfully minimizes steric hindrance at the solid–liquid interface for RgpB recognition, resulting in high sensitivity and operational simplicity as a colorimetric sensor suitable for POCT applications. To assess the storage stability of PIMA‐GcP‐MB‐modified CAL‐LIGHT‐KT, the responses to RgpB (96 nM) were measured after one month of storage under lyophilized conditions at room temperature and water‐moistened conditions at 4°C. No visible coloration of the supernatant was observed during sample preparation prior to the assay, suggesting negligible spontaneous release of MB‐labeled peptide fragments. Furthermore, no change in absorbance was observed (Figure 6c), indicating that long‐term storage is feasible. The high storage stability is likely attributed to the simple response mechanism, in which peptide‐MB fragments were released into the solution from the microsphere precipitate upon peptide cleavage.

Next, we cultured microorganisms known to express or not express gingipain to validate the sensor by measuring the absorbance responses to *P. gingivalis*, the control bacterium *Fusobacterium nucleatum*, and the fungus *Candida albicans*, as well as to α‐amylase, an enzyme present in saliva. Only *P. gingivalis* produced a high absorbance response (Figure 6d), and MALDI‐TOF‐MS analysis of the corresponding solution showed a peak attributed to IDGGC‐MB fragments (Figure 6e). These results confirmed the selectivity toward RgpB secreted by *P. gingivalis*. Additionally, to evaluate the potential effects of the saliva matrix, the absorbance response to RgpB spiked into normal saliva from pooled human donors was evaluated using a 1:1 mixture of saliva and Tris buffer (20 mM, pH 7.6, with 100 mM NaCl, 5 mM CaCl_2_, 1 mM DTT). The buffer was added to adjust the sample to the optimal pH for RgpB activity and to provide a reducing environment required for this cysteine protease. The absorbance response to RgpB in pooled saliva was lower than that observed in buffer alone (Figure S17). The salivary matrix reduced absorbance at 96 nM RgpB (signal) and slightly increased absorbance in the absence of RgpB (background), thereby lowering the signal‐to‐background (S/B) ratio from 21.4 to 7.1. This reduction may be attributed to matrix effects, such as partial desorption of PIMA‐GcP‐MB from the CaCO_3_ surface and reduced accessibility of RgpB to the immobilized peptide substrate. Nevertheless, RgpB‐spiked saliva still produced a clear absorbance response, demonstrating the feasibility of selective RgpB detection in the complex salivary matrix.

Finally, PIMA‐GcP‐MB‐modified CAL‐LIGHT‐KT was applied to the sensing of clinical gingival crevicular fluid (GCF) samples collected from 28 tooth sites in a set of seven subjects with periodontal disease sampled at a dental clinic. The detailed information of the clinical cohort regarding patient demographics and clinical characteristics of the patients with periodontitis (Table S3), as well as site‐specific clinical parameters of GCF collection sites (Table S4), is provided in the Supporting Information. The gingipain activity measured with our method correlated well with the qPCR results (Figure 6f, Pearson's *r* = 0.79; 95% confidence interval [CI], 0.59–0.85). This correlation coefficient is higher than that reported for a fluorescence nanosensor (*r* = 0.71) [11] and a plasmonic colorimetric nanosensor (*r* = 0.55) [8], which also relies on peptide cleavage. To evaluate whether the observed correlation was specific to *P. gingivalis*, we also examined the relationship between the detected gingipain activity and the total bacterial load determined by qPCR (Figure S18a). Although a positive correlation was observed (Pearson's *r* = 0.66; 95% CI, 0.38–0.76), the correlation was weaker than that obtained for *P. gingivalis*. Because the total bacterial load includes *P. gingivalis*, we further analyzed the correlation between the detected gingipain activity and the non‐*P. gingivalis* bacterial load, calculated by subtracting the *P. gingivalis* count from the total bacterial count (Figure S18b). In this case, virtually no correlation was observed (Pearson's *r* = 0.04; 95% CI, −0.34 to 0.23). These results indicate that the correlation between the detected gingipain activity and total bacterial load is primarily attributable to *P. gingivalis*, supporting the conclusion that the absorbance signal predominantly reflects the *P. gingivalis* count.

The superior correlation observed for clinical samples can be attributed to the selectivity of the AGPRID substrate. In the oral environment, host‐derived proteases, including kallikrein‐related peptidases (KLKs) and papain‐family cathepsins, are present and have been reported to increase under inflammatory conditions [50, 51, 52]. While some of these enzymes cleave at the C‐terminal side of arginine, the AGPRID sequence does not match the optimal motifs for KLKs [53, 54] or most cathepsins (e.g., L, V, S, F, or B). Although cathepsin K has been reported to recognize PR motifs similar to AGPRID [55], its potential interference is considered negligible for two primary reasons. First, the reported concentration of cathepsin K in the GCF, even in chronic periodontitis patients, is approximately 0.05 nM [56], which is below the LOD of this work (0.25 nM). Second, cathepsins typically exhibit optimal activity under weakly acidic conditions (around pH 5), whereas GCF is weakly alkaline [57]. These physiological factors minimize the likelihood of false‐positive signals from host‐derived enzymes.

Although the present clinical study demonstrated the feasibility of the proposed assay, the sample size was limited to 28 tooth sites from seven patients. Therefore, the findings should be considered as preliminary evidence of clinical applicability. Further validation with larger and more diverse patient populations will be required to establish diagnostic performance and account for biological variability associated with periodontal disease. Nevertheless, the present findings demonstrate the potential of this system as a simple and robust colorimetric platform for monitoring *P. gingivalis* activity without complex reagent preparation or handling.

## 3 Conclusion

In this study, we developed a simple and robust chromogenic release‐based assay for monitoring *P. gingivalis* activity, which utilizes RgpB‐mediated cleavage of an MB‐modified peptide and surface functionalization of CaCO_3_ microspheres. Peptide cleavages were confirmed even after conjugation of the MB‐modified peptides to PIMA, indicating that the PIMA backbone does not interfere with the enzymatic reactions. In contrast, when the peptides were immobilized on the surface of CaCO_3_ microspheres, the cleaved fragments containing a di‐aspartic acid motif were not released into the supernatant. Furthermore, in the case of RgpB, a reduced enzymatic response was observed when the amount of immobilized PIMA‐peptide‐MB was low. These results provide insight into the mechanisms of enzymatic responses and peptide adsorption at the microsphere interface. Finally, a chromogenic release‐based assay system was successfully established, where RgpB activity induced coloration of the supernatant. The assay also exhibited high storage stability and high selectivity toward *P. gingivalis*. When the responses to clinical GCF samples were compared with qPCR results, a good correlation was observed (Pearson's *r* = 0.79; 95% confidence interval, 0.59–0.85). This work represents an important advance in POCT for colorimetric detection of *P. gingivalis* activity. However, significant practical challenges remain, including the mechanical stability of the surface‐modified dye‐labeled peptide during brushing, compatibility with surfactants, and long‐term shelf life in toothpaste. Future work will focus on integrating this assay into toothpaste formulations to enable routine, at‐home monitoring before the onset of symptoms. This would offer the potential to add pH stabilizers to improve detection sensitivity, but could also present limitations in terms of interferants.

## 4 Experimental Section

Detailed experimental procedures are provided in the Supporting Information.

## Author Contributions

**Zhicheng Jin**: writing – review and editing, methodology. **Jan Potempa**: methodology, writing – review and editing. **Jesse V. Jokerst**: funding acquisition, supervision, writing – review and editing. **Mari Adachi**: investigation, validation. **Natalia O. Tjokro**: investigation. **Lubna Amer**: investigation, writing – review and editing. **Diego F. Trujillo**: investigation, writing – review and editing. **Anthony J. O'donoghue**: investigation, validation. **Yuki Hiruta**: conceptualization, writing – original draft, investigation, funding acquisition, methodology. **Casey Chen**: supervision.

## Ethics Statement

The protocol of GCF collection and testing was approved by the UCSD and USC Institutional Review Boards (HS‐24‐00312) and was in accordance with the ethical guidelines for human subjects research established by the Helsinki Declaration of 1975. There is no clinical study registration number because this is not an NIH‐defined clinical trial, as human subjects were not prospectively assigned to an intervention. Subjects were selected from patients receiving dental treatment at the Herman Ostrow School of Dentistry and provided informed consent.

## Conflicts of Interest

The authors declare no conflicts of interest.

## Supporting information




**Supporting File**: advs77102‐sup‐0001‐SuppMat.pdf.

## Data Availability

The data that support the findings of this study are available from the corresponding author upon reasonable request.
