# Supplementary material for: Protease‐Triggered Chromogenic Release From Peptide‐Modified Calcium Carbonate Microspheres Enables Colorimetric Detection of Porphyromonas gingivalis Activity
Source: Adv Sci (Weinh). 2026 Aug 18;13(58):e77102. doi: 10.1002/advs.77102 (PMC13483131; doi:10.1002/advs.77102)
Supplement: Supplementary file 1 [file ADVS-13-e77102-s001.pdf]

Supporting Information

Protease-Triggered Chromogenic Release from Peptide-Modified Calcium Carbonate Microspheres Enables Colorimetric Detection of *Porphyromonas gingivalis* Activity

Yuki Hiruta,[a,b,*] Zhicheng Jin,[a] Mari Adachi,[c] Lubna Amer,[d] Natalia O. Tjokro,[e] Diego F. Trujillo,[f] Jan Potempa,[g,h] Anthony J. O'Donoghue,[f] Casey Chen,[e] and Jesse V. Jokerst[a,d*]

[a]Aiiso Yufeng Li Family Department of Chemical and Nano Engineering, University of California, San Diego, La Jolla, CA 92093, United States
[b]Department of Applied Chemistry, Faculty of Science and Technology, Keio University, Yokohama, Kanagawa 223-8522, Japan
[c]School of Biological Sciences, Molecular and Cell Biology, University of California San Diego, La Jolla, CA 92093, United States
[d]Program in Materials Science and Engineering Program, University of California, San Diego, La Jolla, CA 92093, United States
[e]Herman Ostrow School of Dentistry, University of Southern California, Los Angeles, CA 92093, United States
[f]Skaggs School of Pharmacy and Pharmaceutical Sciences, University of California, San Diego, La Jolla, CA 92093, United States
[g]Department of Microbiology, Faculty of Biochemistry, Biophysics and Biotechnology, Jagiellonian University, Krakow 30-387, Poland
[h]Department of Oral Immunology and Infectious Diseases, School of Dentistry, University of Louisville, Louisville, Kentucky 40202, United States
***Corresponding authors' emails: hiruta@applc.keio.ac.jp (Y.H.), jjokerst@ucsd.edu (J.V.J.)**

Table of Contents

1. Materials S2
2. Instruments S2
3. Material synthesis and methods S3
 3.1 Peptide synthesis S3
 3.2 Synthesis of peptide-MB conjugates S3
 3.3 Synthesis of PIMA-peptide-MB conjugates S3
 3.4 Gel electrophoresis S4
 3.5 Cleavage of peptides S4
 3.6 Cleavage of peptide-MB conjugates S4
 3.7 Cleavage of PIMA-peptide-MB conjugates S4
 3.8 Modification of PIMA-peptide-MB conjugates onto calcium carbonate microspheres S5
 3.9 Trypsin and RgpB response of PIMA-peptide-MB modified $CaCO_3$ microspheres S5
 3.10 Storage stability of PIMA-GcP-MB modified $CaCO_3$ microspheres S5
 3.11 Microorganisms response of PIMA-peptide-MB modified $CaCO_3$ microspheres S5
 3.12 GCF collection and testing S6
4. References S6
Figure S1. ESI-MS and HPLC data of the synthesized peptides S7
Figure S2. Confirmation of GcP cleavage by RgpB S8
Figure S3. Confirmation of GcPDD cleavage by RgpB S9
Figure S4. ESI-MS and HPLC data of the synthesized peptide-MB conjugates S10
Figure S5. Confirmation of GcP-MB cleavage by RgpB S11
Figure S6. Confirmation of GcPDD-MB cleavage by RgpB S12
Figure S7. Confirmation of PIMA-peptide-MB cleavage by trypsin and RgpB with ESI-MS S13
Figure S8. Confirmation of PIMA-peptide-MB cleavage by trypsin and RgpB with absorbance measurement S14
Figure S9. The morphologies of porous $CaCO_3$ microspheres S15
Figure S10. Colorimetric response of PIMA-peptide-MB modified $CaCO_3$ microspheres to trypsin S16
Figure S11. Confirmation of trypsin response of PIMA-GcP-MB modified $CaCO_3$ microspheres S17
Figure S12. Verification of the adsorption of trypsin-mediated fragment of PIMA-GcPDD-MB onto $CaCO_3$ S18
Figure S13. Evaluation of the effect of $CaCO_3$ on RgpB activity measured by HPLC S19
Figure S14. Quantitative evaluation of PIMA-GcP-MB adsorption on the surface of $CaCO_3$ microspheres S20
Table S1. The adsorption amount of PIMA-GcP-MB depending on the amount of each $CaCO_3$ S21
Table S2. The recovery of peptide-MB released into the supernatant upon RgpB treatment S22
Figure S15. Confirmation of RgpB response of PIMA-GcP-MB modified $CaCO_3$ microspheres S23
Figure S16. RgpB concentration-dependent absorption spectra S24
Figure S17. RgpB response in saliva S25
Table S3. Demographic and clinical characteristics of the patients with periodontal disease S26
Table S4. Site-specific clinical parameters of GCF collection sites S27
Figure S18 Correlation between absorbance variation and bacterial load S28

1. Materials

4-(2-hydroxyethyl)-1-piperazineethanesulfonic acid (HEPES), Trizma® base, Trizma® hydrochloride, DL-dithiothreitol (DTT), trifluoroacetic acid (TFA, HPLC grade, >99%), poly(isobutylene-*alt*-maleic anhydride) (PIMA, M_w ~6,000 Da), Atto MB2 maleimide, triethylamine (TEA), calcium chloride, and trypsin from porcine pancreas were purchased from Sigma Aldrich (St. Louis, MO). Acetonitrile (ACN, HPLC grade), dimethyl sulfoxide (DMSO), and sodium chloride were purchased from Fisher Scientific International, Inc. (Hampton, NH). Fmoc-Rink amide MBHA resin (0.67 mmol/g, 100-150 mesh) were purchased from AAPPTec, LLC (Louisville, KY). Ultrapure water (18 MΩ·cm) was obtained from a Milli-Q Academic water purification system (Millipore Corp., Billerica, MA). Amicon® ultra-0.5 centrifugal filter units (M.W. cutoff =3 kDa) were from MilliporeSigma (St. Louis, MO). CAL-LIGHT-KT, CAL-LIGHT-SA, and PORECAL-N (calcium carbonate microspheres) were kindly provided by Shiraishi Central Laboratories Co., Ltd. (Hyogo, Japan). α-Amylase was purchased from LEE BioSolutions (Maryland Heights, MO). Bacteria: *P. gingivalis* (ATCC 33277, RRID: NCBITaxon_837) and *F. nucleatum* (ATCC 25586, RRID: NCBITaxon_851), Fungi: *C. albicans* (ATCC 90028, RRID: NCBITaxon_5476) were purchased from ATCC (Manassas, VA, USA). Arg-specific gingipain (RgpB) was isolated from *P. gingivalis* as described previously[1], with minor modifications. RgpB was expressed with a C-terminal 8x-his tag in a mutant *P. gingivalis* strain that lacked Lys-specific gingipain (ΔKgp). Normal saliva from pooled human donors (CAT No: 991-05-P) was purchased from Medix Biochemica (Maryland Heights, MO).

2. Instruments

Peptides were synthesized using an automated Eclipse™ peptide synthesizer (AAPPTec, Louisville, KY). All high-performance liquid chromatography (HPLC) purifications and analyses, except for the experiment shown in Figure S13, were performed using a Shimadzu LC-40 HPLC system equipped with a LC-40D solvent delivery module, photodiode array detector SPD-M40, and degassing unit DGU-403. SHIM-PACK GIS C18, 5 μm was used for purification (column size: 100 mm × 22 mm i.d.) and analysis (column size: 100 mm × 4.6 mm i.d.). Synthesis and enzymatic cleavage were confirmed using Electrospray ionization mass spectrometry (ESI-MS) via the Micromass Quattro Ultima mass spectrometer. Matrix-assisted laser desorption/ionization-time-of-flight (MALDI-TOF) mass spectrometer (autoflex maX, BRUKER) with α-cyano-4-hydroxycinnamic acid (HCCA) as the matrix was also used for the confirmation of enzymatic peptide cleavage. NanoDrop One UV-vis spectrometer (Thermo Fisher Scientific) was used for quantification of the peptide concentration. FreeZone Plus 2.5 freeze dry system (Labconco Corp., Kansas City, MO) was used for lyophilization. The optical absorption measurements, except for the experiment shown in Figure S17, were collected using a hybrid multi-mode microplate reader (Synergy™ H1 model, BioTek Instruments, Inc.) in a 96-well plate. Scanning electron microscopy (SEM) images were obtained using the Zeiss Sigma 500 operated at 3 kV with 30 μm aperture. $CaCO_3$ microspheres were drop-cast onto a silicon substrate (Ted Pella Inc.) and sputter-coated with a gold alloy prior to SEM observation.

3. Material synthesis and methods

3.1 Peptide synthesis

Peptides were synthesized through standard solid phase Fmoc synthesis on Rink-amide resin with N-terminal acetylation, following our previous protocol.[2] All peptides were N-terminally acetylated. The peptides were purified by reversed-phase high-performance liquid chromatography (HPLC), identified by the ESI-MS and its purity was confirmed by HPLC. The absorbance at 205 nm of aqueous peptide solution was measured to quantify the peptide concentration. Finally, peptides were lyophilized and stored at room temperature.

3.2 Synthesis of peptide-MB conjugates

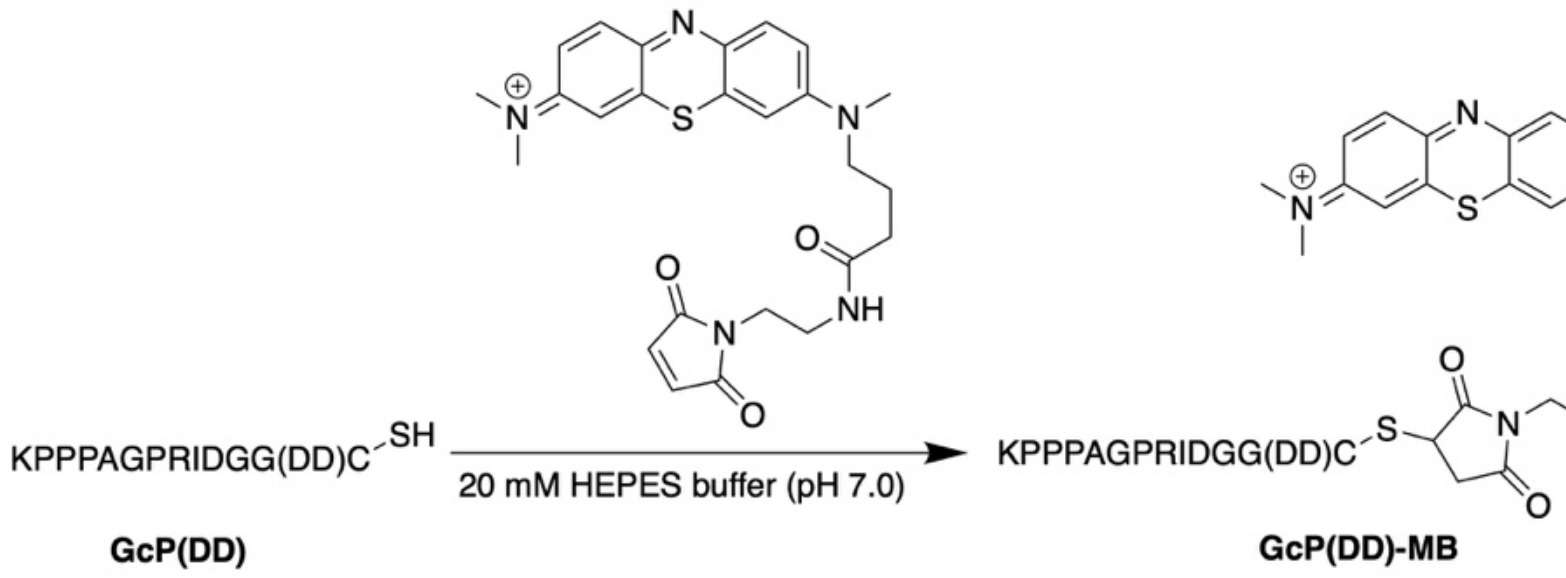

Methylene Blue derivative, Atto MB2 maleimide (0.5 mg) was dissolved in DMSO. Peptides, **GcP(DD)** was dissolved 20 mM HEPES buffer (pH 7.0, 470 μL). These solutions were mixed and shaken at room temperature for 3 h. Crude peptide-MB conjugate was purified by HPLC, eluted at 6.0 mL/min with water/acetonitrile (90/10, v/v) for 1min, followed by the gradient elution from 80/20 (v/v) to 60/40 (v/v) for 39 min. Finally, peptide-MB conjugates were lyophilized and stored at -20 °C. The peptide-MB conjugates were identified by ESI-MS and its purity was confirmed by HPLC. The peptide-dye concentration was calculated by the absorbance at 668 nm ($\varepsilon = 1.1 \times 10^5$ M^{-1} cm^{-1}).

3.3 Synthesis of PIMA-peptide-MB conjugates

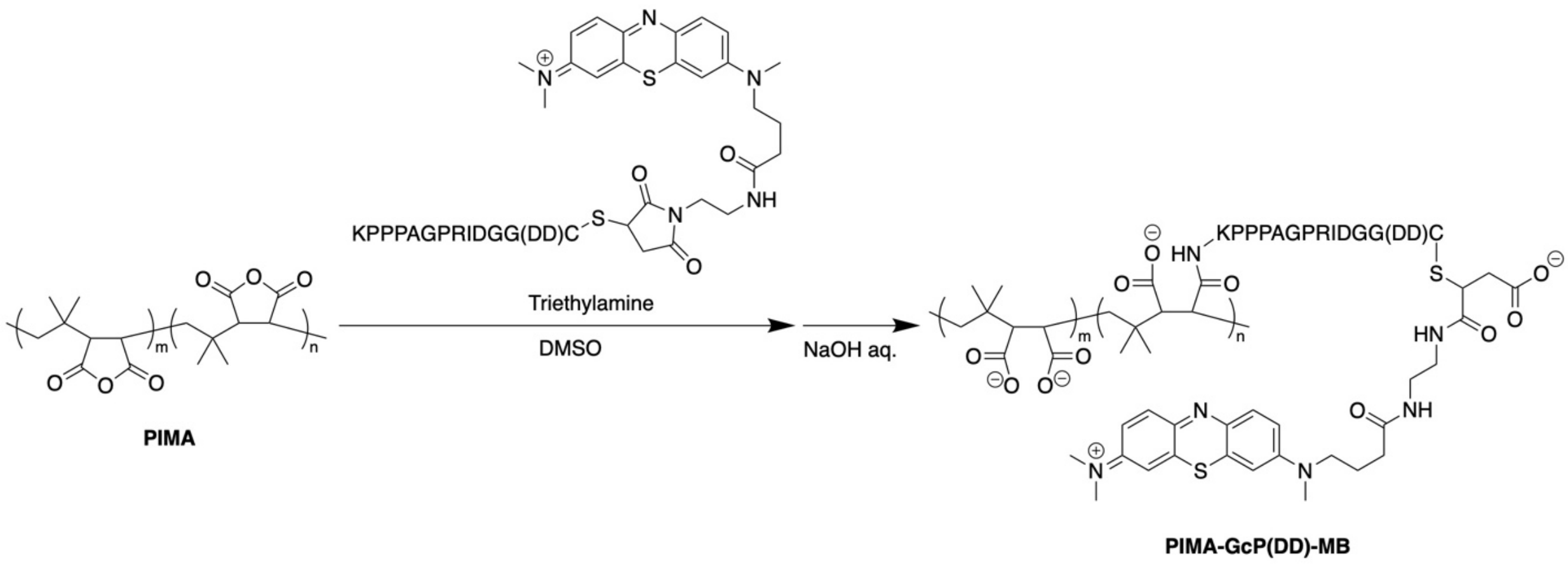

The equivalent was calculated based on the molecular weight of one repeating unit of PIMA, which is 154.2 g/mol. PIMA (10 eq.), **GcP(DD)-MB** (1 eq.), and TEA (3 eq.) were added to DMSO (100 μL), and shaken at 60 °C for 2 days. Water (1.9 mL) was added to the reaction solution. 1 M NaOH aqueous solution was added dropwise to hydrolyze the unreacted maleic anhydride, and succinimidyl thioether

moiety. The hydrolysis was stopped when the pH test paper indicated a weak alkaline condition (pH 8-9). The reaction solution was loaded into Amicon® ultra-0.5 centrifugal filter units (M.W. cutoff = 3 kDa) and centrifuged at 14,000 × g to purify and concentrate the sample. Concentrated sample was washed three times with water by repeated dilution and centrifugation. The purified PIMA-conjugated peptide-MB solutions were stored in water as a stock solution until further use. The PIMA-conjugated peptide-MB concentrations, expressed as the molar concentration of peptide-dye units (not PIMA-peptide-MB conjugates) were calculated by the absorbance at 668 nm ($\varepsilon = 1.1 \times 10^5$ M^{-1} cm^{-1}).

3.4 Gel electrophoresis

Gel electrophoresis measurement was conducted on a 0.7% (w/v) agarose gel prepared in 100 mL of TBE buffer (1 × strength, 89 mM tris, 89 mM boric acid, pH 8.3, with 1 mM EDTA). The aliquots (15 µL) of the peptide-MB (GcP-MB and GcPDD-MB), and PIMA-peptide-MB (PIMA-GcP-MB and PIMA-GcPDD-MB) were mixed with 5 µL of glycerol, homogenized then loaded into the gel wells, and run at 6.5 V/cm for 20 min. The white-light image was taken by a smartphone in a light box.

3.5 Cleavage of peptides

GcP or GcPDD solutions (0.5 mg/mL) in Tris buffer (20 mM, pH 7.6, with 100 mM NaCl, 5 mM $CaCl_2$, 1 mM DTT) were incubated with RgpB (192 nM) at 37 °C for 2 h. The mixture was then injected to HPLC system. The fractions were collected, and the molecular weight was determined with the ESI mass spectrometer.

3.6 Cleavage of peptide-MB conjugates

GcP-MB or GcPDD-MB solutions (10 µM) in Tris buffer (20 mM, pH 7.6, with 100 mM NaCl, 5 mM $CaCl_2$, 1 mM DTT) were incubated with RgpB (192 nM) at 37 °C for 2 h. The mixture was then injected to HPLC system. The fraction was collected, and the molecular weight was determined with the ESI mass spectrometer.

3.7 Cleavage of PIMA-peptide-MB conjugates

PIMA-GcP-MB or PIMA-GcPDD-MB solutions (6 µM as MB concentration) in Tris buffer (20 mM, pH 7.6, with 100 mM NaCl, 5 mM $CaCl_2$, 1 mM DTT) were incubated with trypsin (2 µM) or RgpB (192 nM) at 37 °C for 2 h. Absorption spectra of the mixtures were measured with microplate reader in a 96-well plate. The mixture was then injected to HPLC system. The fraction was collected, and the molecular weight was determined with the ESI mass spectrometer.

3.8 Modification of PIMA-peptide-MB conjugates onto calcium carbonate microspheres

1.5, 0.8, or 0.4 mg of calcium carbonate microspheres (CAL-LIGHT-KT, CAL-LIGHT-SA, or PORECAL-N) were suspended in 50 µL water. The stock solution of PIMA-GcP(DD)-MB was added to the suspension to obtain a final amount of 1 nmol, calculated as the amount of MB. This protocol was designed for a single assay, and the amounts were scaled up proportionally when multiple assays were performed. The suspension was shaken for 1 h and then centrifuged at 10,000 × g for 2 min, and the supernatant was removed to eliminate unbound PIMA-GcP(DD)-MB. The precipitate was washed repeatedly with the same volume of water two times and then with tris buffer once, each time followed by centrifugation at 10,000 × g for 2 min and removal of the supernatant.

3.9 Trypsin and RgpB response of PIMA-peptide-MB modified $CaCO_3$ microspheres

PIMA-GcP(DD)-MB modified $CaCO_3$ microspheres in Tris buffer (20 mM, pH 7.6, with 100 mM NaCl, 5 mM $CaCl_2$, 1 mM DTT) were incubated with trypsin or RgpB for 2 h. After natural sedimentation of the microspheres, the white-light image was taken by a smartphone in a light box. Subsequently, the sample was centrifuged at 10,000 × g for 2 min, and the absorption spectra of supernatant was measured with microplate reader in a 96-well plate. The molecular weight of the peptide-MB released into the supernatant was identified by MALDI-TOF mass spectrometer.

3.10 Storage stability of PIMA-GcP-MB modified $CaCO_3$ microspheres

The PIMA-GcP-MB-modified CAL-LIGHT-KT prepared as described in Protocol 3.8 was suspended in water. Half of the suspension was lyophilized and stored at room temperature, while the remaining suspension was stored in water at 4 °C. After one month of storage, the lyophilized sample was redispersed in water. An aliquot of each suspension was transferred to a reaction tube and centrifuged at 10,000 × g for 2 min. The supernatant was visually inspected to confirm the absence of blue coloration derived from released PIMA-GcP-MB. After removal of the supernatant, the microspheres were resuspended in RgpB solution, and the response to RgpB was evaluated using the same procedure as describe in Protocol 3.9.

3.11 Microorganisms response of PIMA-peptide-MB modified $CaCO_3$ microspheres

Bacteria: *P. gingivalis* (ATCC 33277) and *F. nucleatum* (ATCC 25586), Fungi: *C. albicans* (ATCC 90028) were cultured, and prepared, following our previous protocol.[3] Colorimetric analysis and identification of the molecular weight of peptide-MB were performed using the same procedure as describe in Protocol 3.9.

3.12 GCF collection and testing

This study protocol received approval from the UCSD and USC Institutional Review Boards (HS-24-00312) and was in accordance with the ethical guidelines for human subjects research established by the Helsinki Declaration of 1975. Subjects were selected from patients receiving dental treatment at the Herman Ostrow School of Dentistry and provided informed consent. Subjects were diagnosed as periodontitis Stage II and III, according to the 2017 World Workshop on the Classification of Periodontal and Peri-implant Diseases and Conditions via extra- and intraoral examination, periodontal probing/examination (clinical attachment level (CAL), tooth mobility, bleeding on probing, gingival biotype), and radiography. Samples were collected, following our previous protocol.[4] Each 30 µL sample was mixed with 30 µL Tris buffer (20 mM, pH 7.6, with 100 mM NaCl, 5 mM $CaCl_2$, 1 mM DTT) and PIMA-GcP-MB modified CAL-LIGHT-KT microspheres and then incubated at 37 °C for 2 h. An additional 140 µL of Tris buffer was added, and the sample was centrifuged at 10,000 × g for 2 min, and the absorption spectra of supernatant was measured with microplate reader in a 96-well plate. The *P. gingivalis* count in GCF was determined by qPCR, following our previous protocol.[3-4] Similar to the *P. gingivalis* count, total bacterial load in the sample was also determined through qPCR using universal primer pair, 5'-GATTAGATACCCTGGTAGTCCAC-3' (forward) and 5'-TACCTTGTTACGACTT-3' (reverse). A standard curve was generated using a mixture of genomic DNA from six bacterial species (*A. actinomycetemcomitans* D7S-1, *S. parasanguinis*, *F. nucleatum*, *A. israelii*, *S. gordonii*, and *P. gingivalis*). Sample Ct values were then converted to estimated cell numbers using this standard curve.

4. References

[1] F. Veillard, B. Potempa, Y. Guo, M. Ksiazek, M. N. Sztukowska, J. A. Houston, L. Koneru, K.-A. Nguyen, J. Potempa, *Biol. Chem.* **2015**, *396*, 377-384.

[2] L. Amer, M. Retout, Z. Jin, S. Kakanar, J. V. Jokerst, *Aggregate* **2025**, *6*, e677.

[3] C. Moore, Y. Cheng, N. Tjokro, B. Zhang, M. Kerr, M. Hayati, K. C. J. Chang, N. Shah, C. Chen, J. V. Jokerst, *Angew. Chem. Int. Ed.* **2022**, *61*, e202201843.

[4] M. Retout, L. Amer, W. Yim, M. N. Creyer, B. Lam, D. F. Trujillo, J. Potempa, A. J. O'Donoghue, C. Chen, J. V. Jokerst, *ACS Nano* **2023**, *17*, 17308-17319.

Table and Figures

ESI-MS and HPLC data of the synthesized peptides

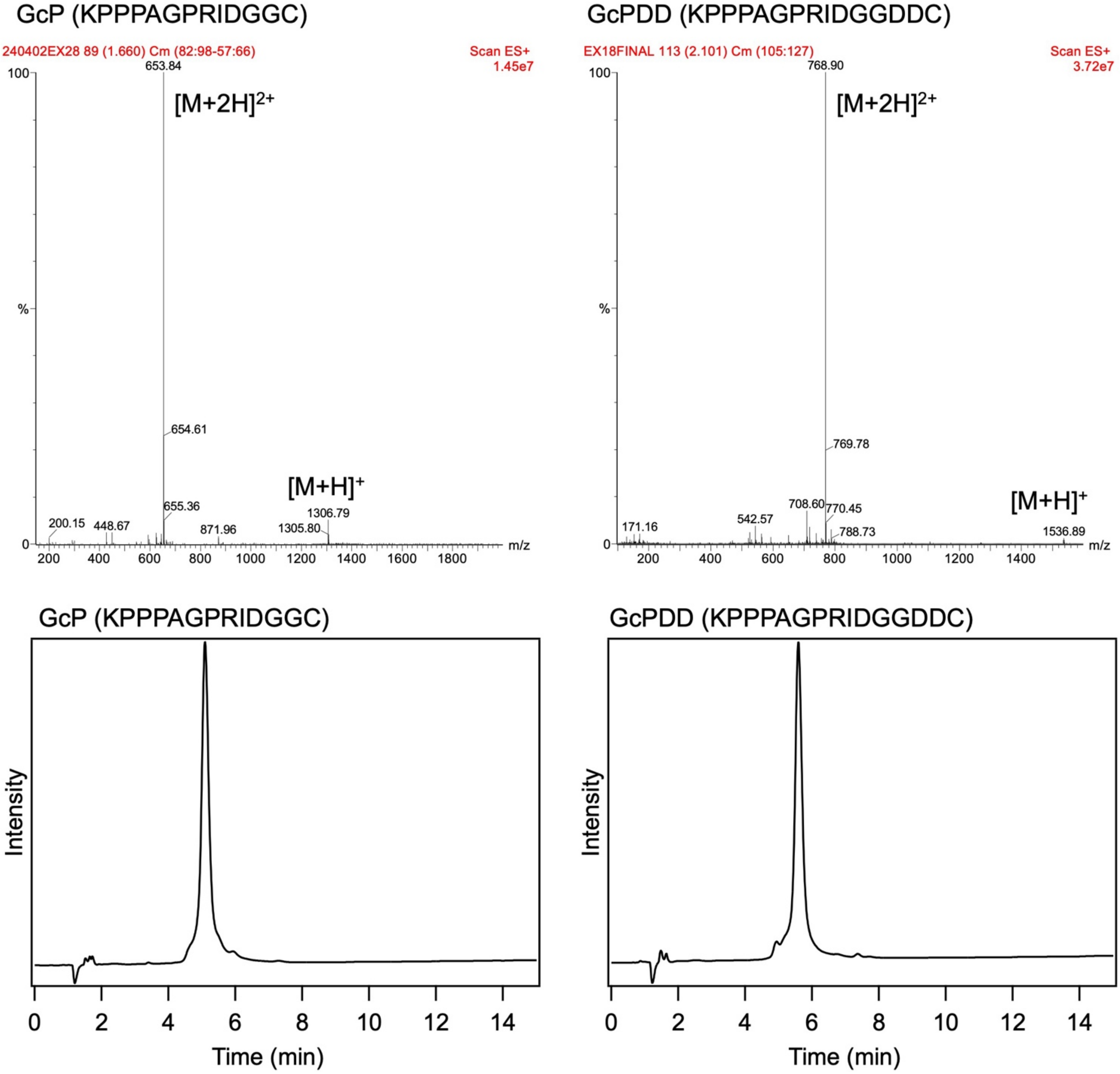

Figure S1. The MS spectra in the positive ion mode are shown in the upper panel. ESI-MS (quadrupole) GcP calcd for $C_{56}H_{94}N_{18}O_{16}S$ $[M+2H]^{2+}$ *m/z* 653.3, found 653.8; GcPDD calcd for $C_{64}H_{104}N_{20}O_{22}S$ $[M+2H]^{2+}$ *m/z* 768.4, found 768.9. The chromatograms are shown in the lower panel. Analytical conditions: 0.05% TFA water/acetonitrile starting with 90/10 (v/v) followed by gradient elution to 80/20 (v/v) for 15 min, 1.0 mL/min, 20 µL injection of 0.5 mg/mL peptide, detected 220 nm. These results confirm that GcP and GcPDD were synthesized with high purity.

Confirmation of GcP cleavage by RgpB

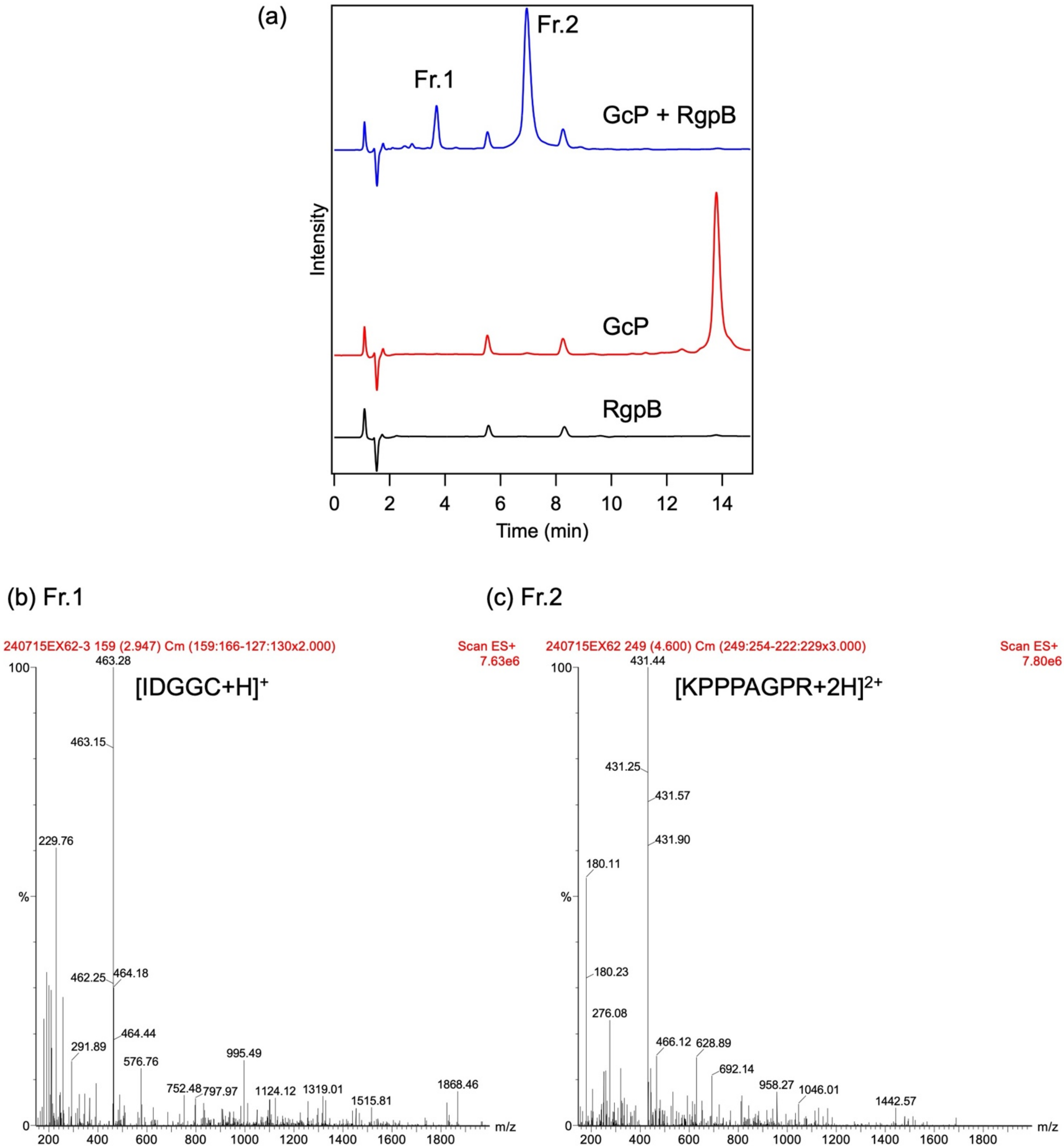

Figure S2. (a) Chromatograms of GcP incubated with RgpB (192 nM), as well as control samples of GcP and RgpB. Analytical conditions: 0.05% TFA water/acetonitrile starting with 95/5 (v/v) followed by gradient elution to 85/15 (v/v) for 15 min, 1.0 mL/min, 20 µL injection of 0.5 mg/mL, detected 220 nm. The MS spectra in the positive ion mode of (b) Fr.1 and (c) Fr.2 from the chromatogram of Figure S2a. ESI-MS (quadrupole) IDGGC calcd for $C_{17}H_{31}N_6O_7S$ $[M+H]^+$ *m/z* 463.2, found 463.3; KPPPAGPR calcd for $C_{39}H_{66}N_{12}O_{10}$ $[M+2H]^{2+}$ *m/z* 431.3, found 431.4. These results confirm that cleavage of GcP occurred at the C-terminal side of arginine (R), as designed.

Confirmation of GcPDD cleavage by RgpB

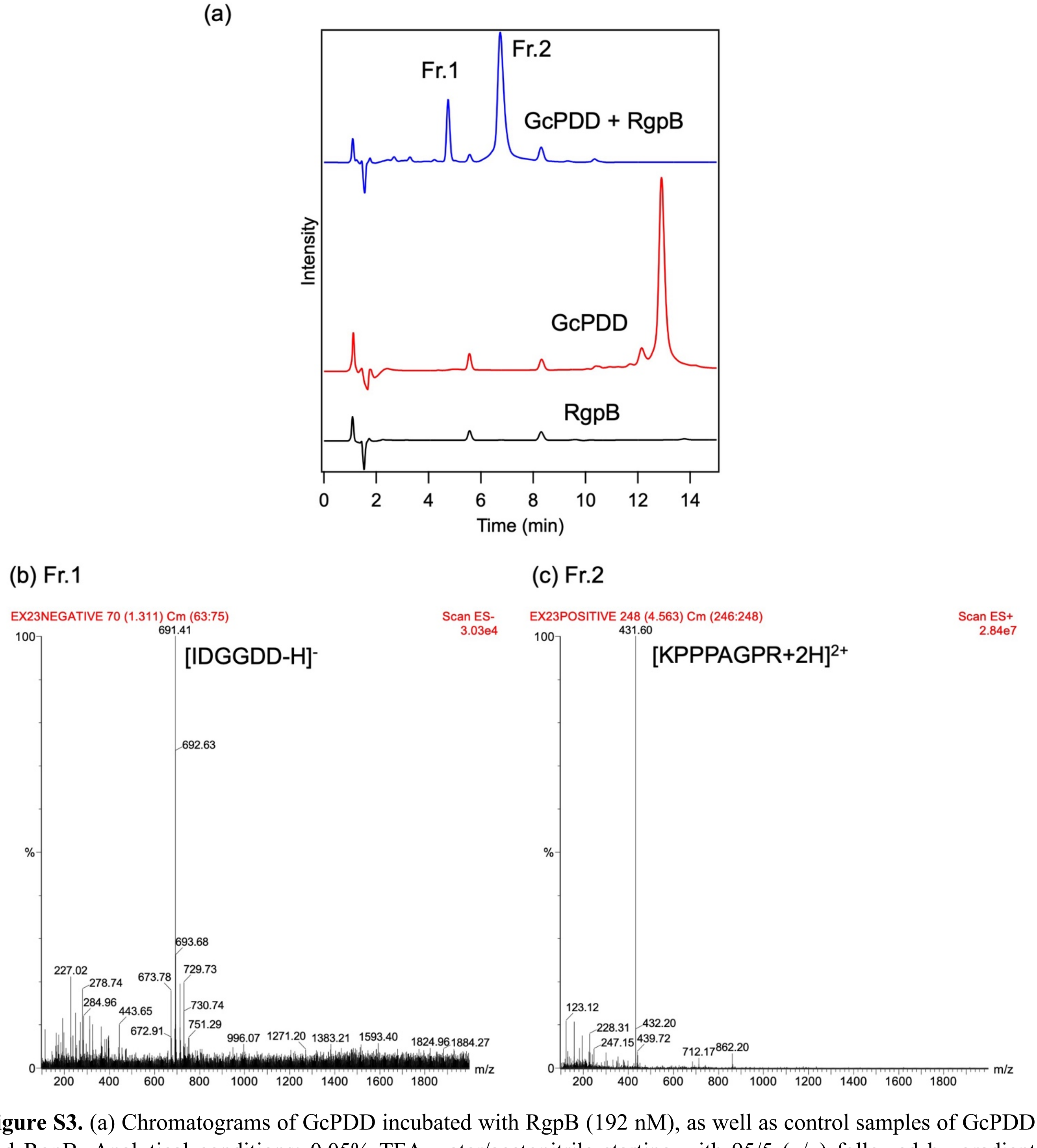

Figure S3. (a) Chromatograms of GcPDD incubated with RgpB (192 nM), as well as control samples of GcPDD and RgpB. Analytical conditions: 0.05% TFA water/acetonitrile starting with 95/5 (v/v) followed by gradient elution to 85/15 (v/v) for 15 min, 1.0 mL/min, 20 µL injection of 0.5 mg/mL GcPDD, detected 220 nm. The MS spectra of (b) Fr.1 in the negative ion mode and (c) Fr.2 in the positive ion mode from the chromatogram of Figure S3a. ESI-MS (quadrupole) IDGGDDC calcd for $C_{25}H_{39}N_8O_{13}S$ $[M-H]^-$ *m/z* 691.2, found 691.4; KPPPAGPR calcd for $C_{39}H_{66}N_{12}O_{10}$ $[M+2H]^{2+}$ *m/z* 431.3, found 431.6. These results confirm that cleavage of GcP occurred at the C-terminal side of arginine (R), as designed.

ESI-MS and HPLC data of the synthesized peptide-MB conjugates

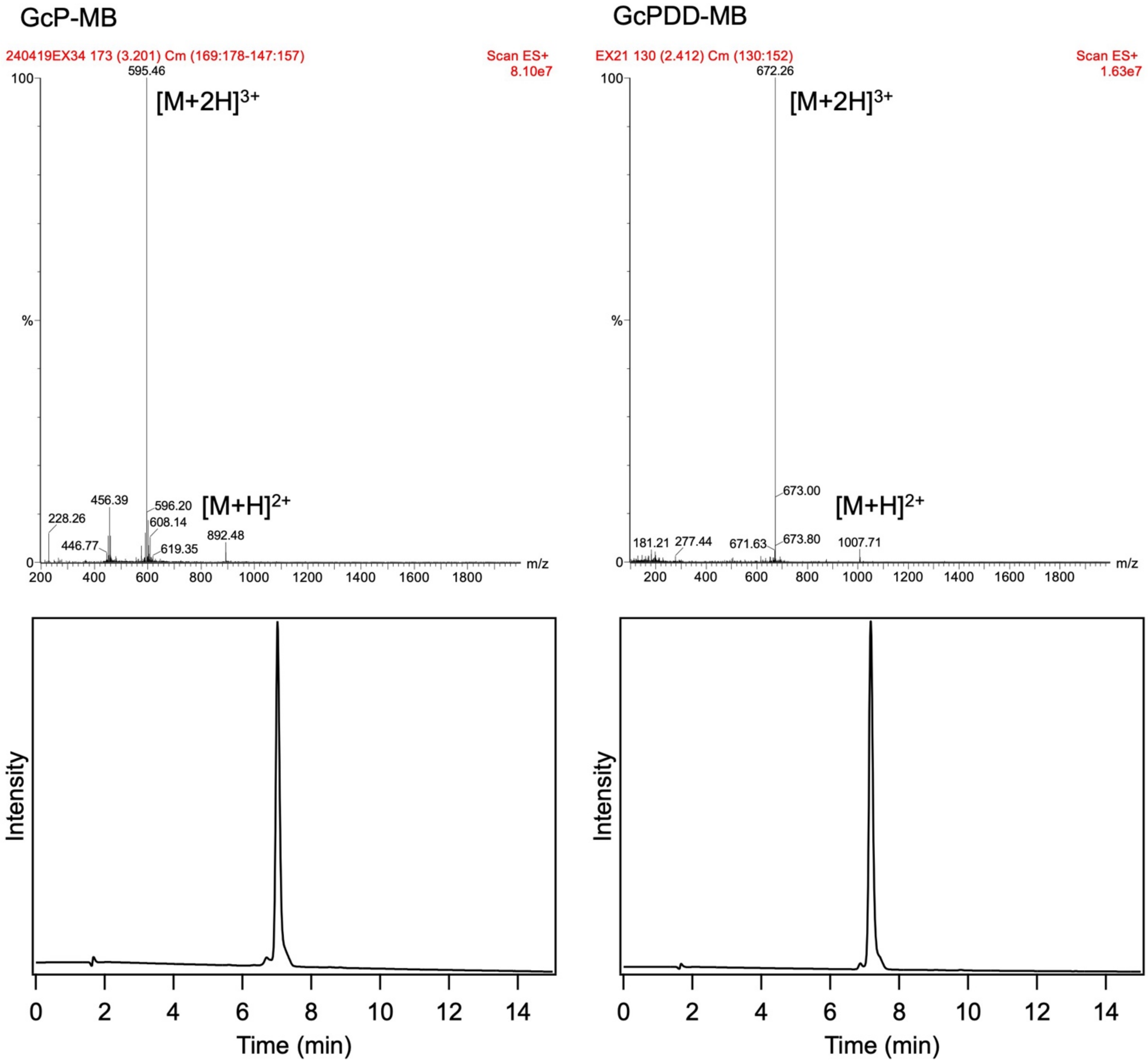

Figure S4. The MS spectra in the positive ion mode are shown in the upper panel. ESI-MS (quadrupole) GcP-MB calcd for $C_{81}H_{122}N_{23}O_{19}S_2$ $[M+2H]^{3+}$ *m/z* 595.0, found 595.5; GcPDD-MB calcd for $C_{89}H_{132}N_{25}O_{25}S_2$ $[M+2H]^{3+}$ *m/z* 671.6, found 672.3. The chromatograms are shown in the lower panel. Analytical conditions: 0.05% TFA water/acetonitrile starting with 90/10 (v/v) followed by gradient elution to 60/40 (v/v) for 15 min, 1.0 mL/min, 20 µL injection of 10 µM peptide-MB conjugates, detected 650 nm. These results confirm that GcP-MB and GcPDD-MB were synthesized with high purity.

Confirmation of GcP-MB cleavage by RgpB

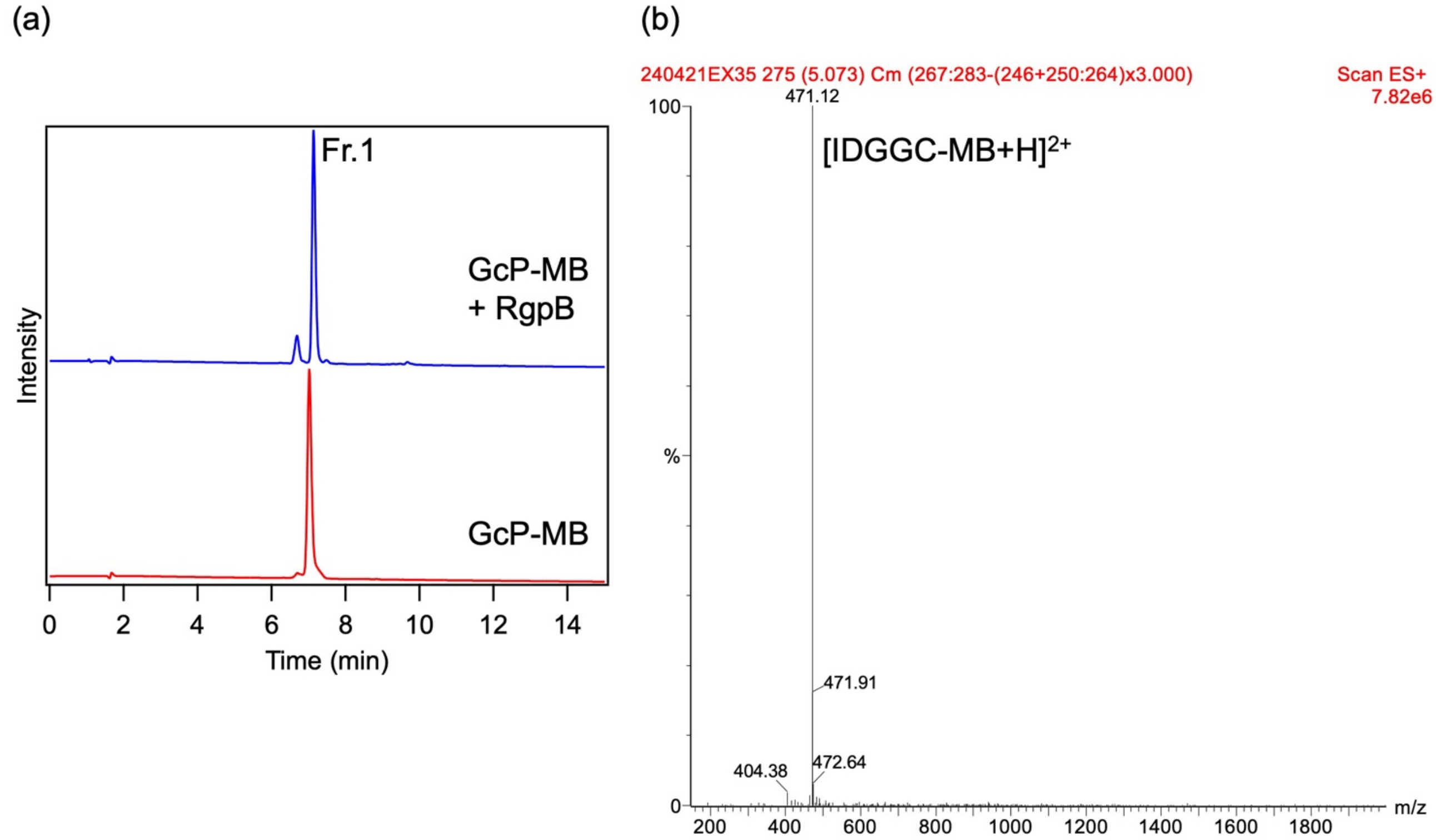

Figure S5. (a) Chromatograms of GcP-MB incubated with RgpB (192 nM), as well as control samples of GcP-MB. Analytical conditions: 0.05% TFA water/acetonitrile starting with 90/10 (v/v) followed by gradient elution to 60/40 (v/v) for 15 min, 1.0 mL/min, 20 µL injection of 10 µM GcP-MB, detected 650 nm. (b) The MS spectra in the positive ion mode of Fr.1 from the chromatogram of Figure S5a. ESI-MS (quadrupole) IDGGC-MB calcd for $C_{42}H_{59}N_{11}O_{10}S_2$ $[M+2H]^{2+}$ *m/z* 470.7, found 471.1. These results confirm that cleavage of GcP-MB occurred at the C-terminal side of arginine (R), as designed.

Confirmation of GcPDD-MB cleavage by RgpB

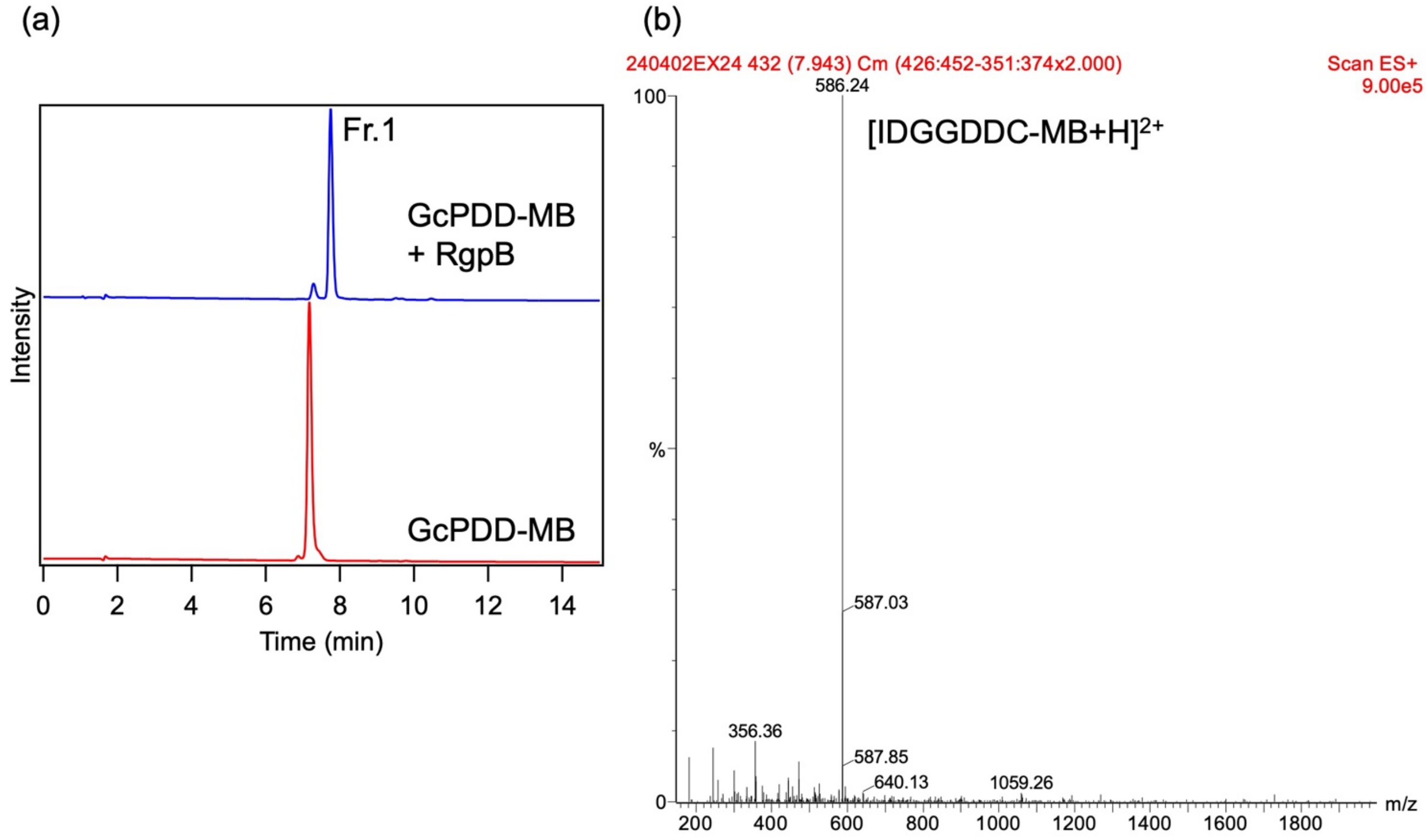

Figure S6. (a) Chromatograms of GcPDD-MB incubated with RgpB (192 nM), as well as control samples of GcPDD-MB. Analytical conditions: 0.05% TFA water/acetonitrile starting with 90/10 (v/v) followed by gradient elution to 60/40 (v/v) for 15 min, 1.0 mL/min, 20 µL injection of 10 µM GcPDD-MB, detected 650 nm. (b) The MS spectra in the positive ion mode of Fr.1 from the chromatogram of Figure S6a. ESI-MS (quadrupole) IDGGDDC-MB calcd for $C_{50}H_{69}N_{13}O_{16}S_2$ $[M+2H]^{2+}$ *m/z* 585.7, found 586.2. These results confirm that cleavage of GcPDD-MB occurred at the C-terminal side of arginine (R), as designed.

Confirmation of PIMA-peptide-MB cleavage by trypsin and RgpB with ESI-MS

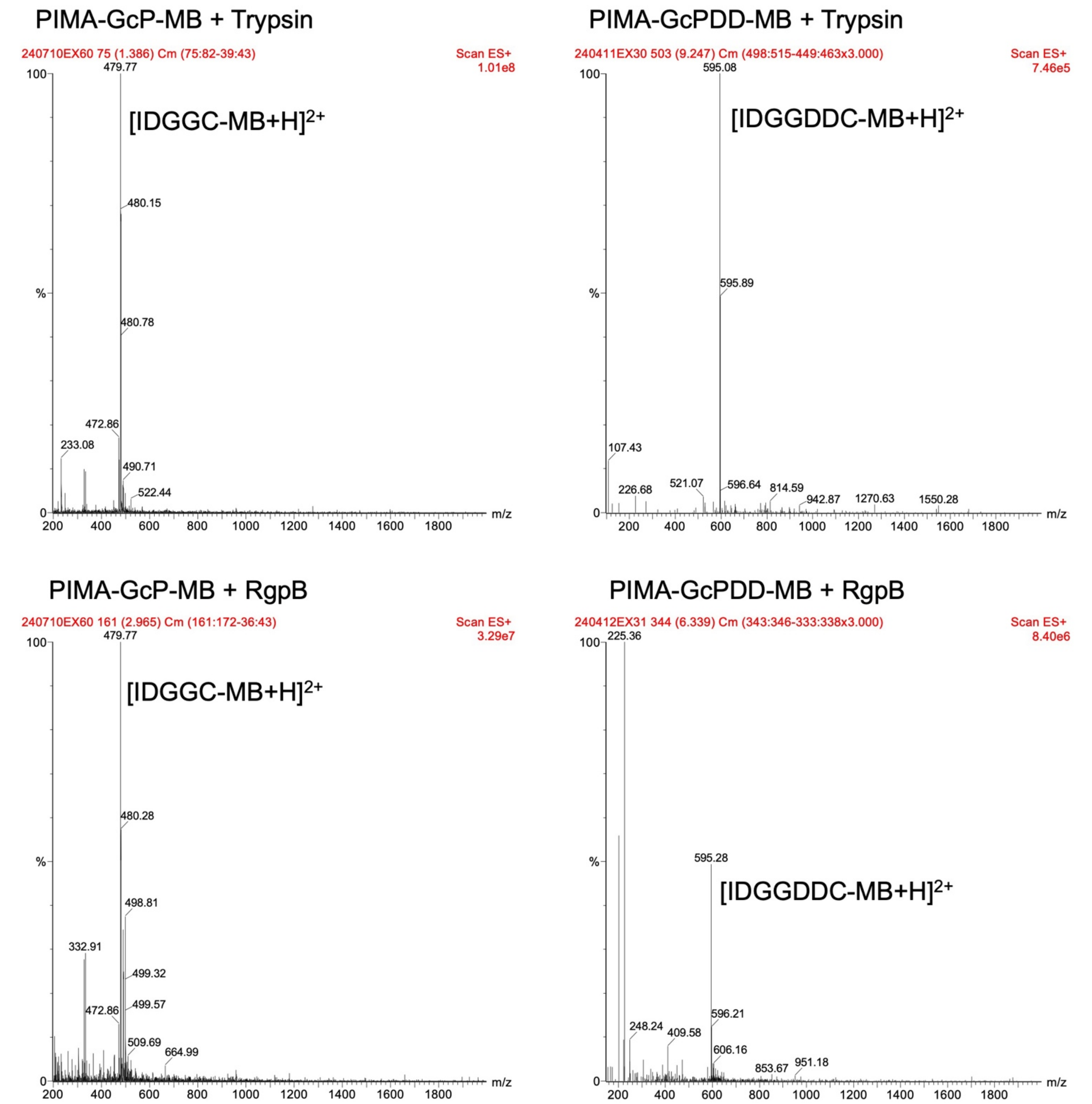

Figure S7. The MS spectra in the positive ion mode of PIMA-GcP(DD)-MB in response to trypsin and RgpB from the chromatogram of Figure 3a, and b in the main text. ESI-MS (quadrupole) IDGGC-MB calcd for $C_{42}H_{61}N_{11}O_{11}S_2$ $[M+H]^{2+}$ *m/z* 479.7, found 479.8 for PIMA-GcP-MB + Trypsin; 479.8 for PIMA-GcP-MB + RgpB. IGDDGGC-MB calcd for $C_{50}H_{71}N_{13}O_{17}S_2$ $[M+H]^{2+}$ *m/z* 594.7, found 595.1 for PIMA-GcPDD-MB + Trypsin; 595.3 for PIMA-GcPDD-MB + RgpB. These results confirm that cleavage occurred at the same site even when the peptide was conjugated to PIMA, indicating that conjugation to PIMA did not interfere with enzyme recognition.

Confirmation of PIMA-peptide-MB cleavage by trypsin and RgpB with absorbance measurement

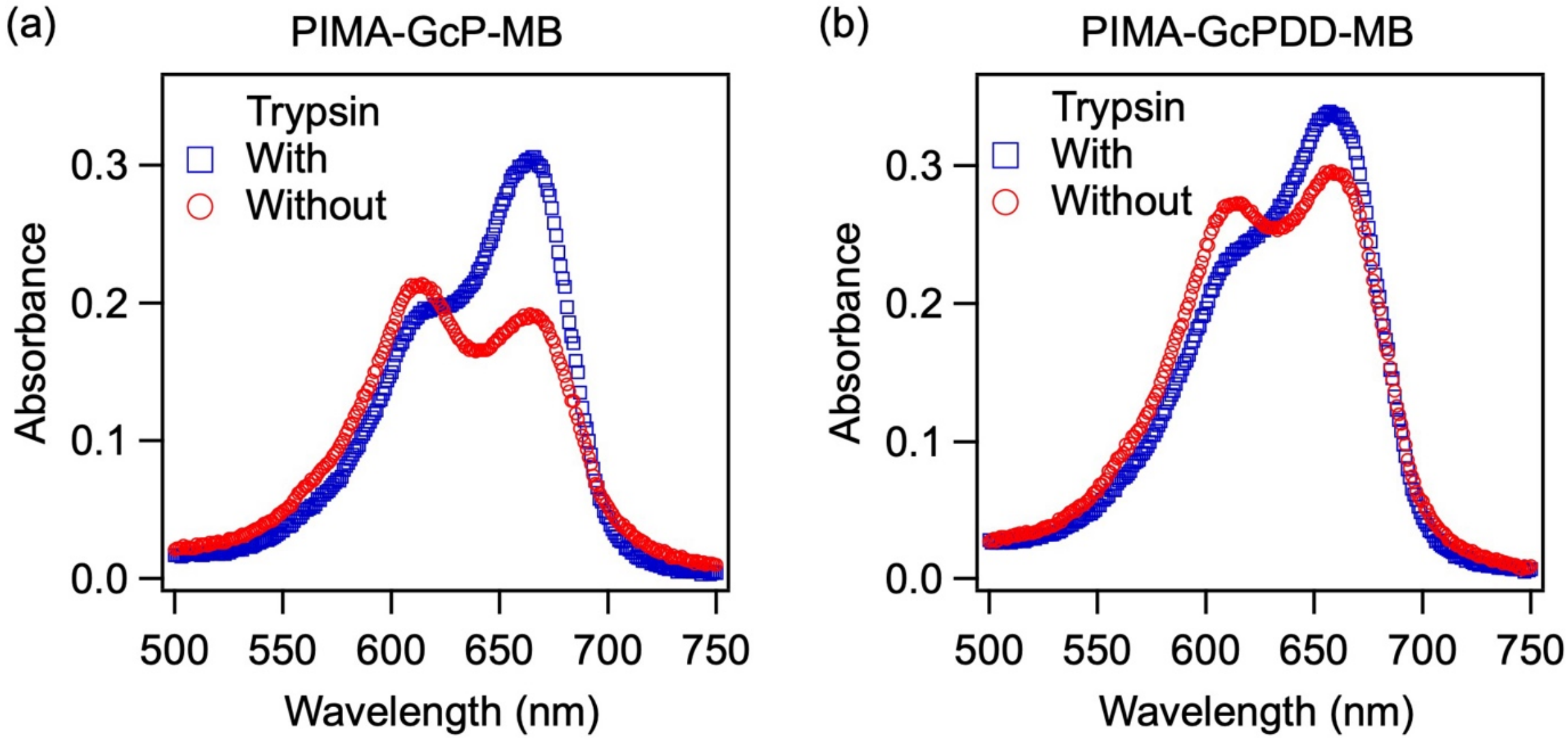

Figure S8. (a, b) Absorption spectra of PIMA-GcP(DD)-MB (6 μM) treated with(out) trypsin (2 μM). All spectra were measured in Tris buffer (20 mM, pH 7.6, with 100 mM NaCl, 5 mM $CaCl_2$, 1 mM DTT). These results confirm that trypsin-mediated cleavage released MB-labeled peptides from the PIMA backbone.

The morphologies of porous $CaCO_3$ microspheres

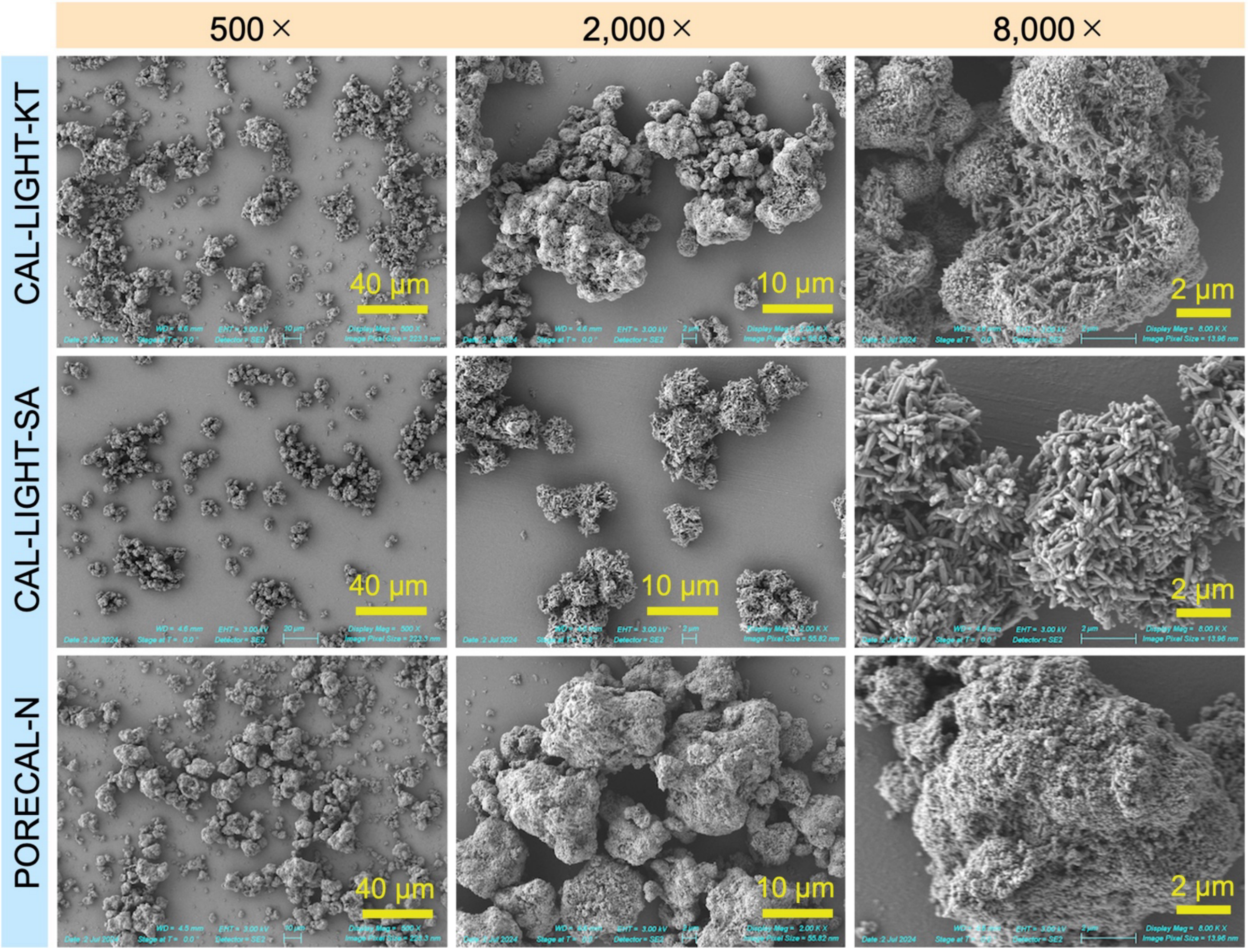

Figure S9. SEM images of porous $CaCO_3$ microspheres (CAL-LIGHT-KT, CAL-LIGHT-SA, and PORECAL-N) at different magnifications.

Colorimetric response of PIMA-peptide-MB modified $CaCO_3$ microspheres to trypsin

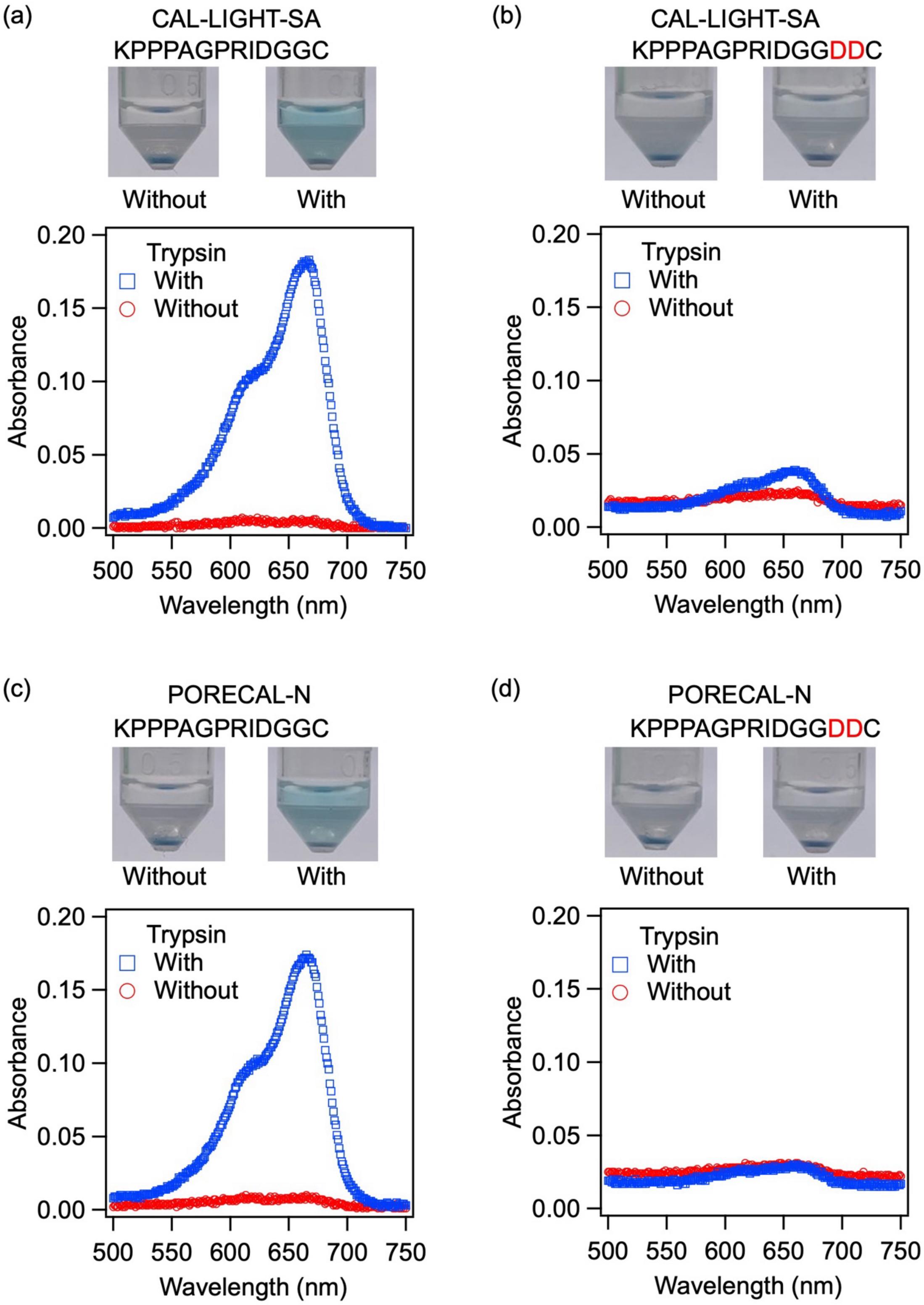

Figure S10. Images of sample solutions with microspheres (1.5 mg) after natural sedimentation post-response to trypsin, and absorption spectra of the supernatants of (a) PIMA-GcP-MB modified CAL-LIGHT-SA, (b) PIMA-GcPDD-MB modified CAL-LIGHT-SA, (c) PIMA-GcP-MB modified PORECAL-N, and (d) PIMA-GcPDD-MB modified PORECAL-N. These results confirm that trypsin cleavage of PIMA-GcP-MB on $CaCO_3$ microspheres releases MB-labeled peptide fragments into the supernatant, whereas fragments derived from PIMA-GcPDD-MB remain adsorbed on the microsphere surface.

Confirmation of trypsin response of PIMA-GcP-MB modified $CaCO_3$ microspheres

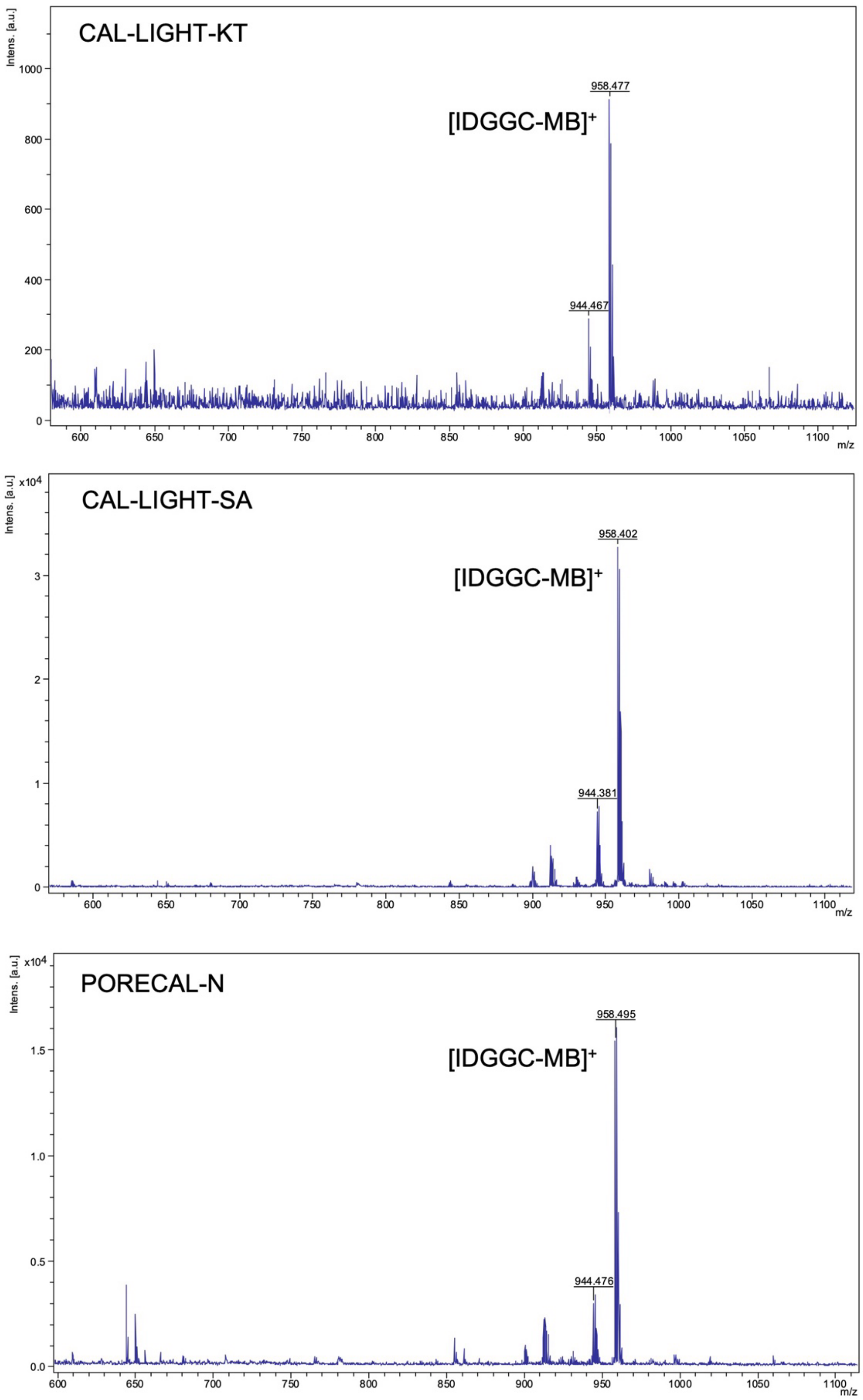

Figure S11. MALDI-MS spectra of the supernatants of PIMA-GcP-modified $CaCO_3$ microspheres after treatment with trypsin from the supernatant of Figure 4a in the main text, and Figure S10a and c in the supporting information. MALDI/TOF-MS IDGGC-MB calcd for $C_{42}H_{60}N_{11}O_{11}S_2$ $[M]^+$ *m/z* 958.4, found 958.5 for CAL-LIGHT-KT; 958.4 for CAL-LIGHT-SA; 958.5 for PORECAL-N. These results confirm that the peptide was selectively recognized and cleaved by trypsin even on the surface of the $CaCO_3$ microspheres.

Verification of the adsorption of trypsin-mediated fragment of PIMA-GcPDD-MB onto $CaCO_3$

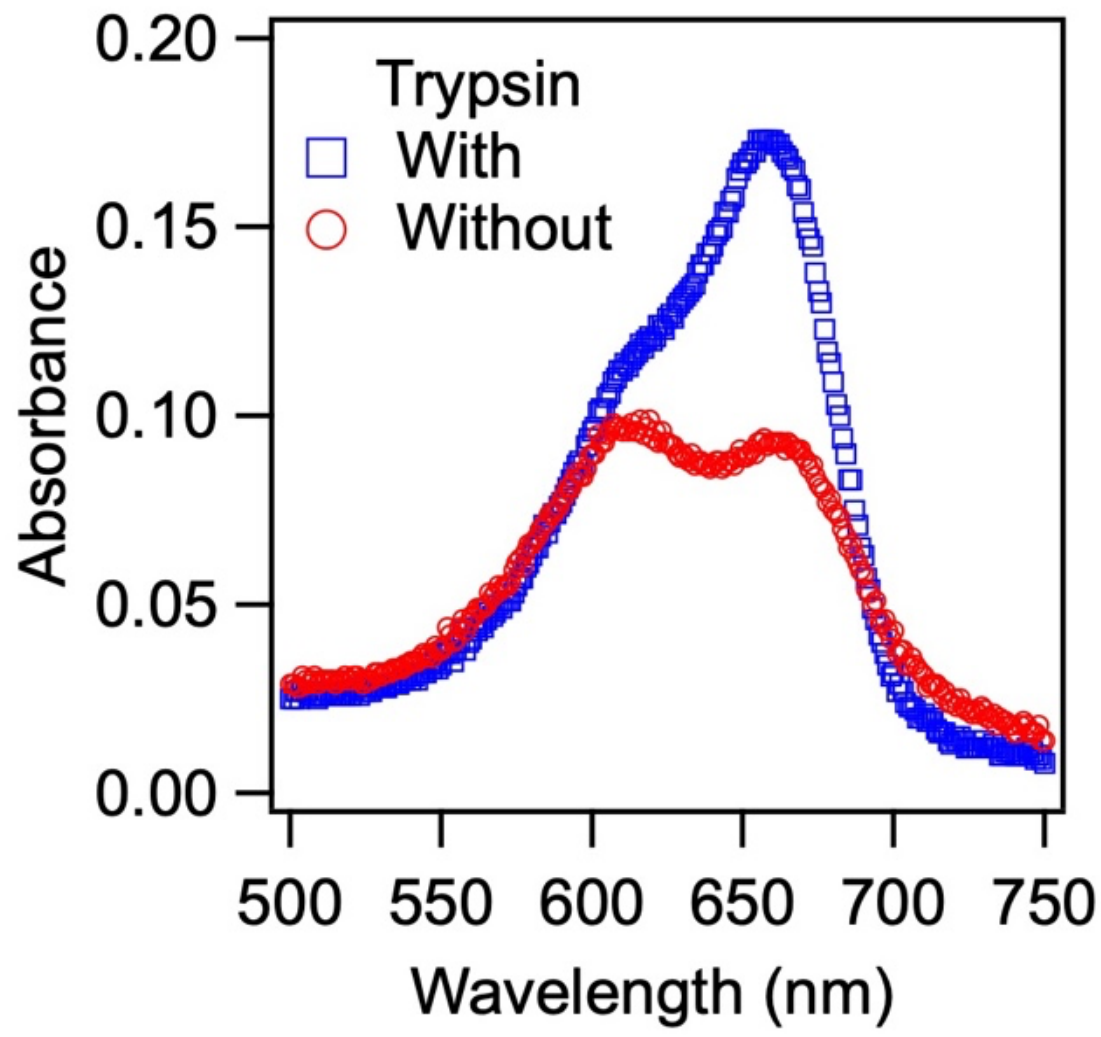

Figure S12. Absorption spectra of PIMA-GcPDD-MB-modified CAL-LIGHT-KT samples dissolved with hydrochloric acid. The samples shown in Figure 4b in the main text were washed to remove the supernatant followed by the addition of 40 µL of 1 M HCl to dissolve $CaCO_3$. The samples were then diluted with140 µL of water, and the absorption spectra were measured with microplate reader in a 96-well plate. These results confirm that although PIMA-GcPDD-MB underwent trypsin-mediated cleavage on $CaCO_3$ surface, the released IDGGDDC-MB fragment strongly adsorbed onto $CaCO_3$.

Evaluation of the effect of $CaCO_3$ on RgpB activity measured by HPLC

300 µL RgpB solution (96 nM) in Tris buffer (20 mM, pH 7.6, with 100 mM NaCl, 5 mM $CaCl_2$, 1 mM DTT) was mixed with 2.3 mg CAL-LIGHT-KT. After centrifugation at 10,000 × g for 2 min, 190 µL of supernatant was collected. As a control, 190 µL of untreated RgpB solution was prepared under the same conditions. GcP stock solution (10 mg/mL) was added to both the collected supernatant and the untreated RgpB solution to obtain a final GcP concentration of 0.5 mg/mL. The mixtures were incubated at 37 °C for 2 h and subsequently analyzed by HPLC. HPLC analyses were performed using Shimadzu LC-2050C 3D equipped with an Inertsil ODS-3 column, (5 µm, 150 mm × 4.6 mm i.d.).

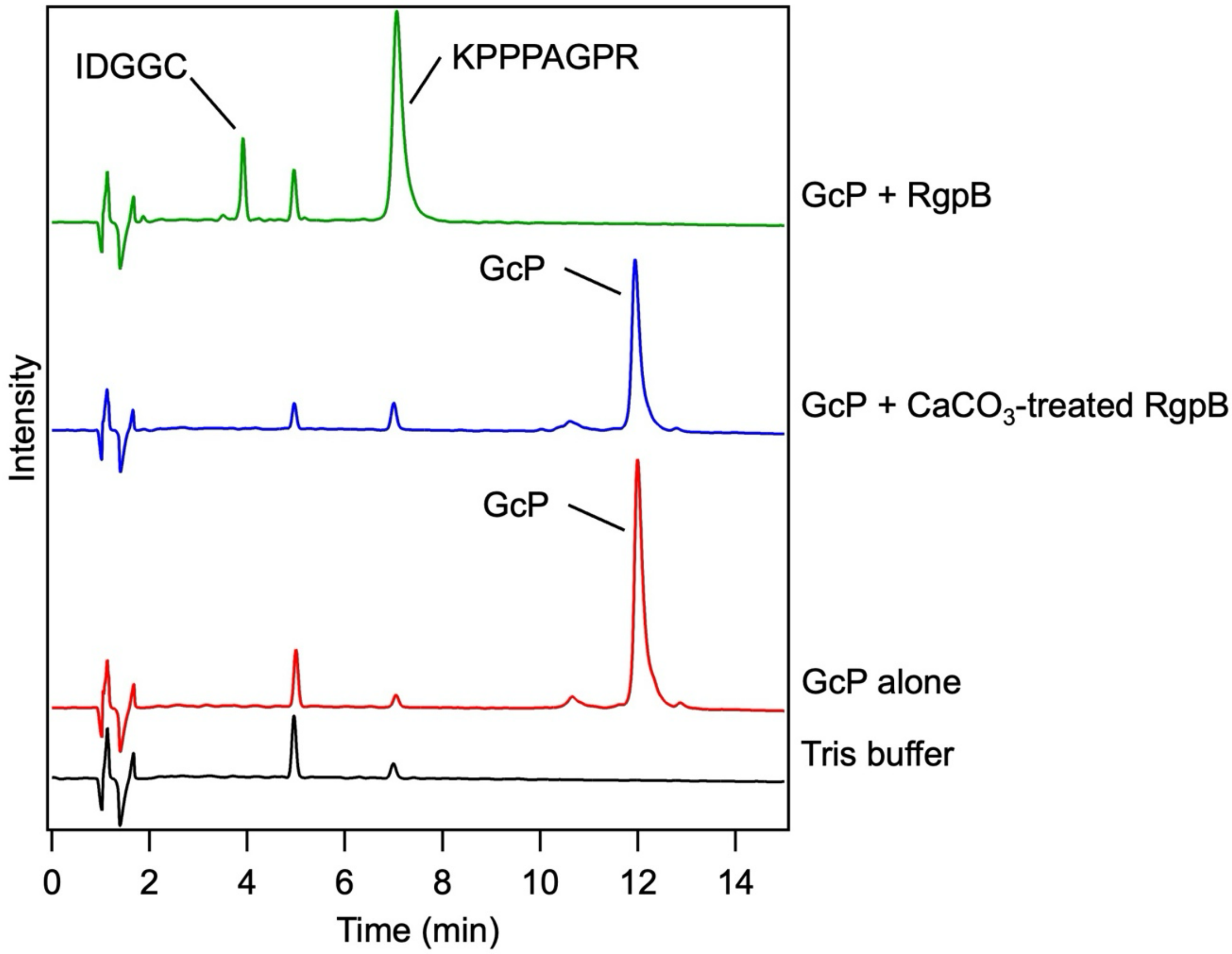

Figure S13. Chromatograms of GcP alone, GcP incubated with RgpB (96 nM), and GcP incubated with the supernatant obtained after incubation of RgpB (96 nM) with $CaCO_3$ microspheres. Analytical conditions: 0.1% TFA water/acetonitrile starting with 95/5 (v/v) followed by gradient elution to 80/20 (v/v) for 15 min, 1.0 mL/min, 20 µL injection of 0.5 mg/mL GcP, detected 220 nm.

Quantitative evaluation of PIMA-GcP-MB adsorption on the surface of $CaCO_3$ microspheres

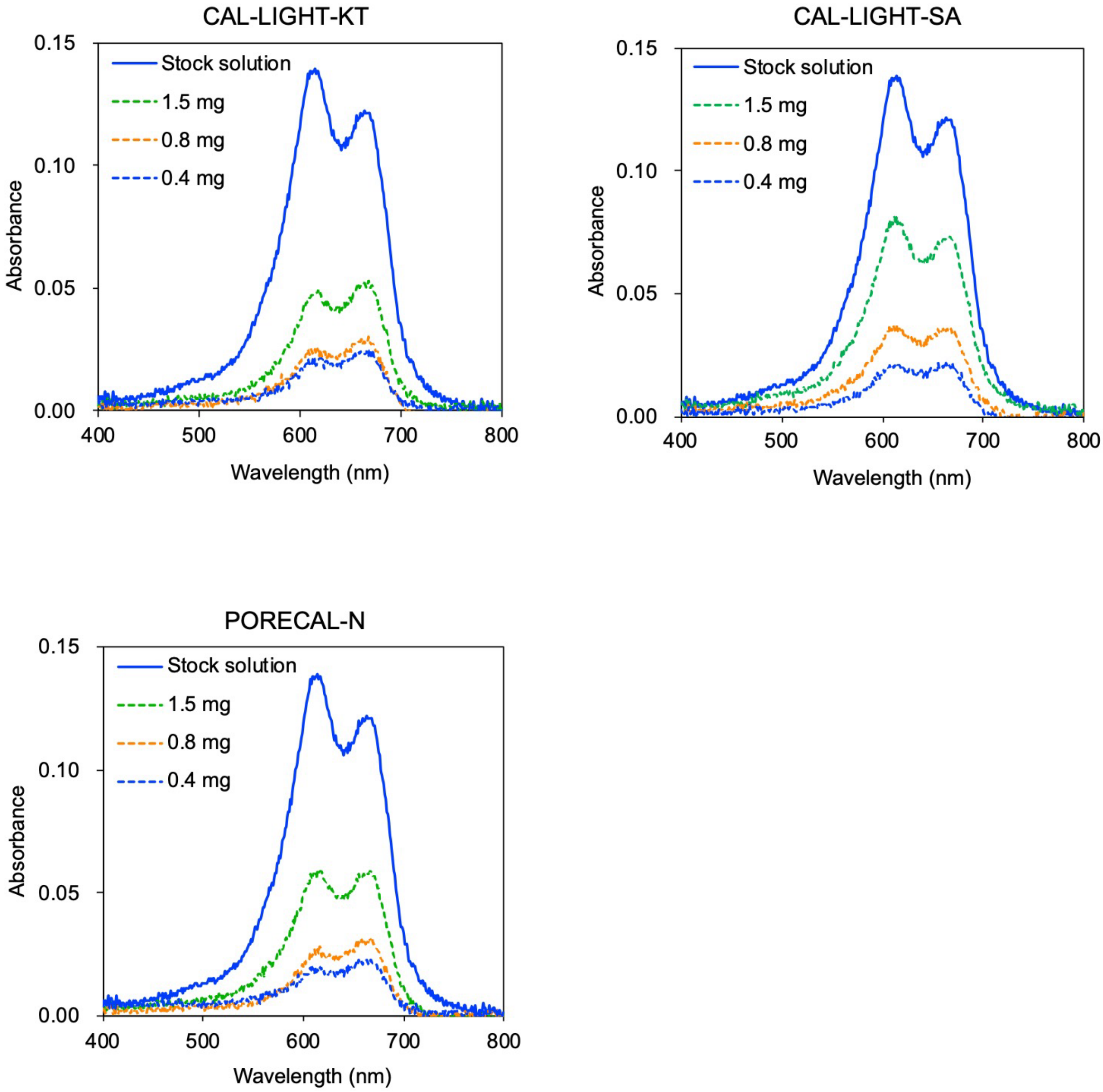

Figure S14. Absorption spectra of the PIMA–GcP–MB solution before adding $CaCO_3$ microspheres (stock solution) and of the supernatants after adding each amount of $CaCO_3$ microspheres.

Table S1. The adsorption amount of PIMA-GcP-MB depending on the amount of each $CaCO_3$

Sample		polymer-to-$CaCO_3$ feed ratio (w/w%)	Polymer-to-$CaCO_3$ adsorption ratio (w/w%)	Surface density of MB on $CaCO_3$ (molecules/nm^2)	surface density of maleic acid units on $CaCO_3$ (molecules/nm^2)
CAL-LIGHT-KT	1.5 mg	0.17	0.14	0.012	0.12
	0.8 mg	0.34	0.26	0.023	0.23
	0.4 mg	0.68	0.39	0.036	0.36
CAL-LIGHT-SA	1.5 mg	0.17	0.14	0.026	0.26
	0.8 mg	0.34	0.24	0.043	0.43
	0.4 mg	0.68	0.28	0.050	0.50
PORECAL-N	1.5 mg	0.17	0.14	0.013	0.13
	0.8 mg	0.34	0.25	0.023	0.23
	0.4 mg	0.68	0.35	0.032	0.32

The concentration of MB was calculated from the difference in absorbance at 668 nm between the stock solution and the solution after adding $CaCO_3$ shown in Figure S14. Since one GcP-MB molecule is conjugated to every 10 repeating units of PIMA, the molecular of PIMA-GcP-MB on the surface was calculated from the MB concentration and the molecular weight of 3,326 g/mol (corresponding to $PIMA_{10}$-GcP-MB per MB molecule). Because the absorption spectra in the Figure S14 include a dimer peak, the absorbance values were corrected using the ratio of absorbance at 668 nm before and after RgpB treatment (Figure 3d in the main text), and the calculations were performed using the molar extinction coefficient of MB ($\varepsilon = 1.1 \times 10^5$).

Table S2. The recovery of peptide-MB released into the supernatant upon RgpB treatment in Figure 5 in the main text relative to the total amount adsorbed on the $CaCO_3$ surface. These results confirm that increasing polymer-to-$CaCO_3$ ratio enhanced the RgpB response across all three types of microspheres.

**Sample**		**Recovery* (%)**
CAL-LIGHT-KT	1.5 mg	12
	0.8 mg	19
	0.4 mg	79
CAL-LIGHT-SA	1.5 mg	6
	0.8 mg	29
	0.4 mg	84
PORECAL-N	1.5 mg	14
	0.8 mg	42
	0.4 mg	84

*The recovery was determined by comparing the absorbance at 668 nm in Figure 5 with the difference in absorbance at 668 nm between the stock solution and the solution after adding $CaCO_3$ shown in Figure S14. Because the absorption spectra in the Figure S14 include a dimer peak, the absorbance values were corrected using the ratio of absorbance at 668 nm before and after RgpB treatment (Figure 3d in the main text).

Confirmation of RgpB response of PIMA-GcP-MB modified $CaCO_3$ microspheres

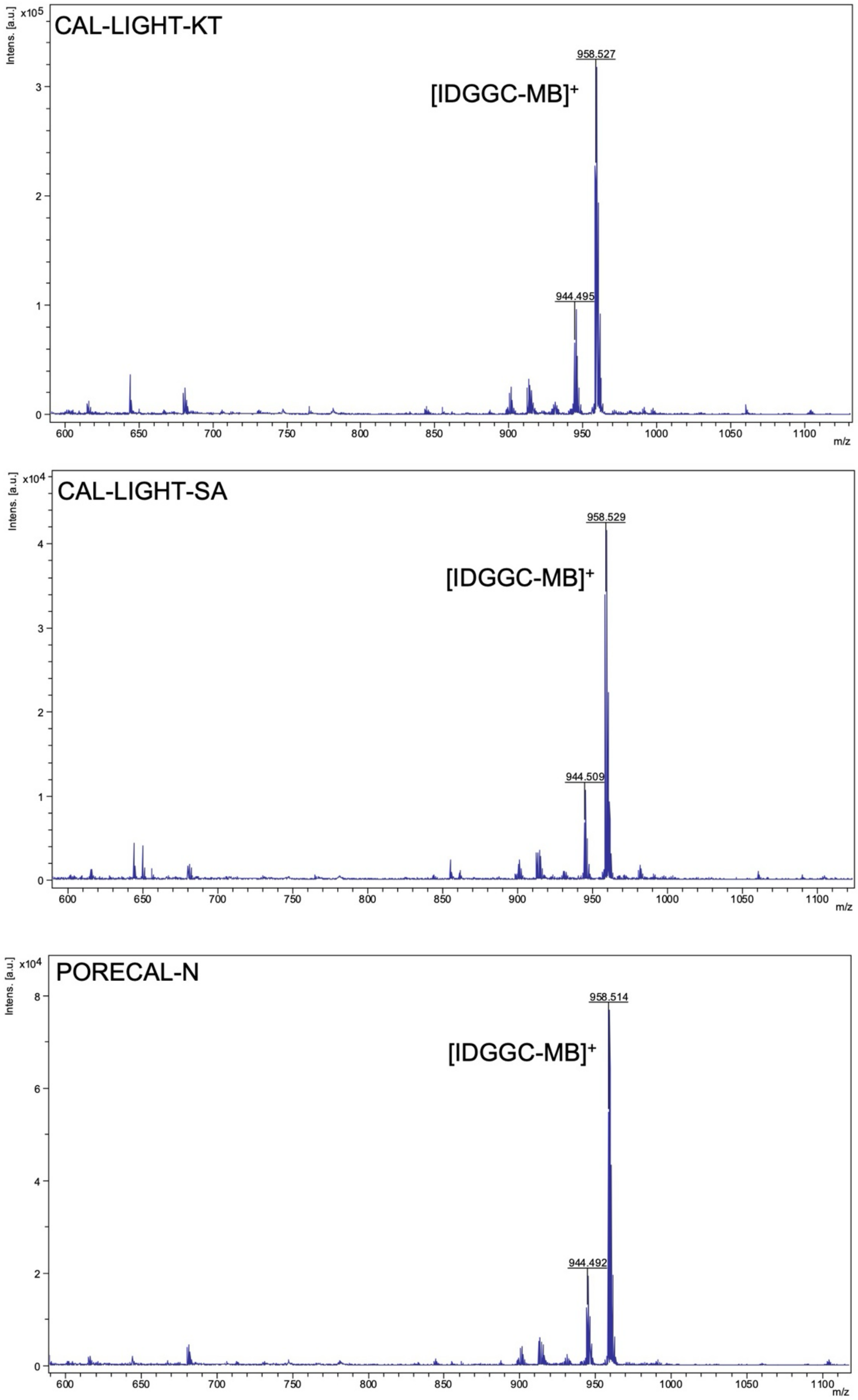

Figure S15. MALDI-MS spectra of the supernatants of PIMA-GcP-modified $CaCO_3$ microspheres after treatment with RgpB from the supernatants of Figure 5 in the main text when the amount of $CaCO_3$ microsphere is 0.4 mg. MALDI/TOF-MS IDGGC-MB calcd for $C_{42}H_{60}N_{11}O_{11}S_2$ $[M]^+$ *m/z* 958.4, found 958.5 for CAL-LIGHT-KT; 958.5 for CAL-LIGHT-SA; 958.5 for PORECAL-N. These results confirm that the peptide was selectively recognized and cleaved by RgpB even on the surface of the $CaCO_3$ microspheres.

RgpB concentration-dependent absorption spectra

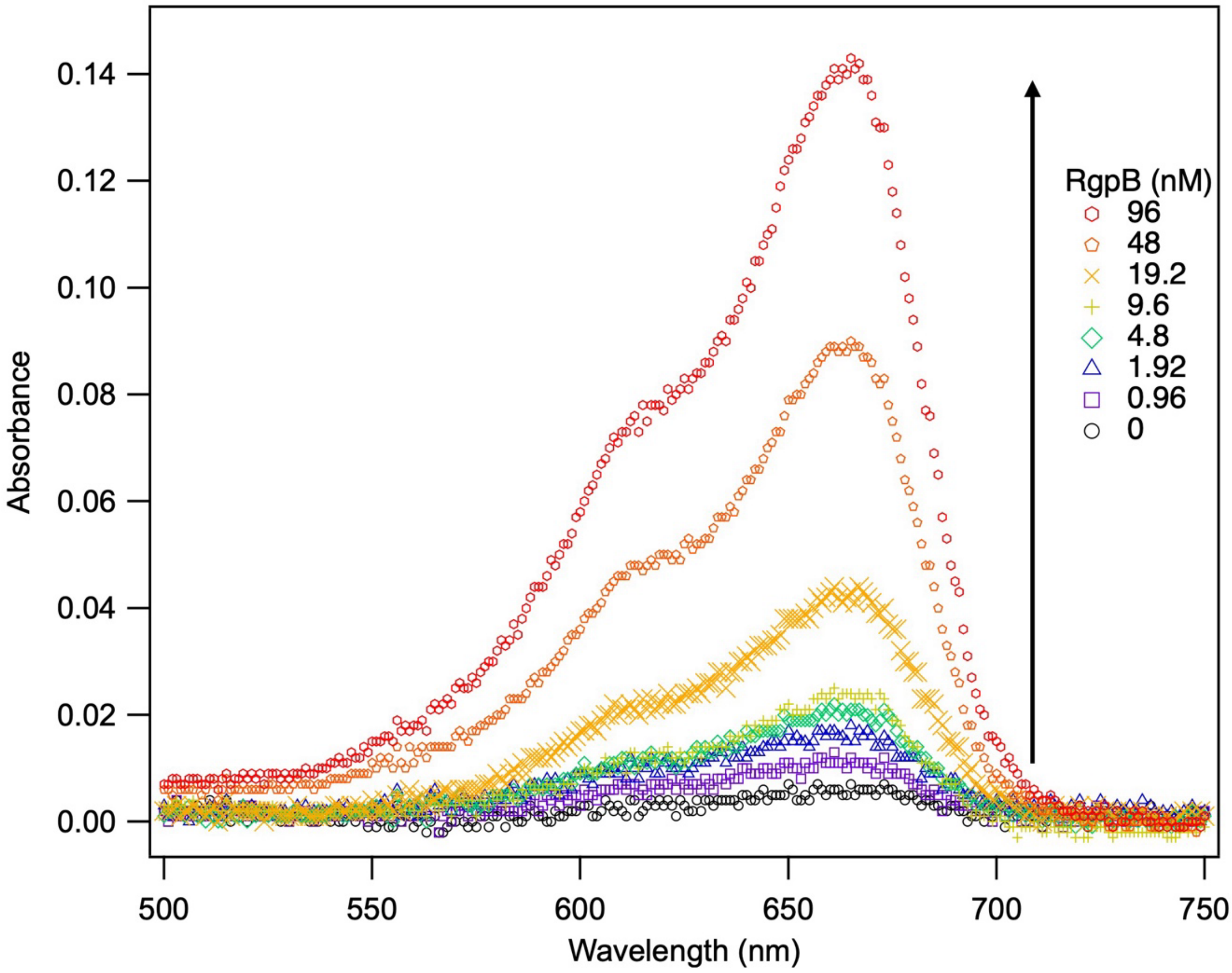

Figure S16. Absorption spectra of the supernatants after treating PIMA-GcP-MB with each concentration of RgpB in Tris buffer (20 mM, pH 7.6, with 100 mM NaCl, 5 mM $CaCl_2$, 1 mM DTT). These results confirm a concentration-dependent increase in absorbance with increasing RgpB concentration.

RgpB response in saliva

PIMA-GcP-MB-modified $CaCO_3$ microspheres were incubated for 2 h with or without RgpB in a 1:1 mixture of normal saliva from pooled human donors and Tris buffer (20 mM, pH 7.6, with 100 mM NaCl, 5 mM $CaCl_2$, and 1 mM DTT). The sample was centrifuged at 10,000 × g for 2 min, and the absorption spectra of supernatant was measured with microplate reader (Infinite 200 PRO, Tecan, Männedorf, Switzerland) in a 96-well plate.

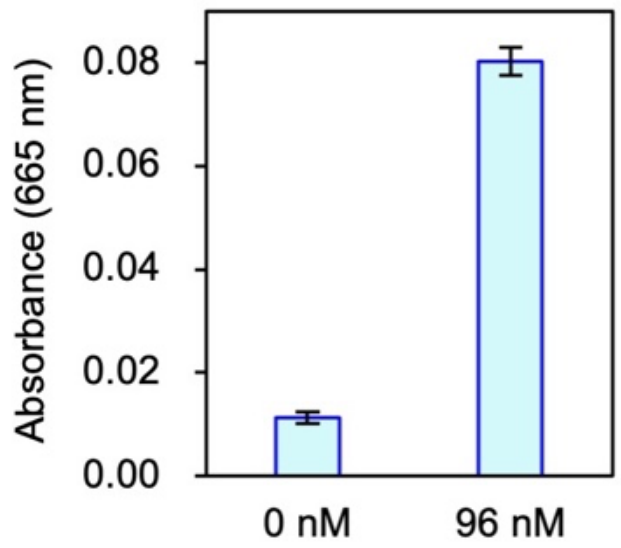

Figure S17. Colorimetric response of PIMA-GcP-MB modified $CaCO_3$ microspheres to RgpB in a mixture of normal saliva from pooled human donors and Tris buffer (20 mM, pH 7.6, with 100 mM NaCl, 5 mM $CaCl_2$, and 1 mM DTT). The error bars represent the standard deviation of three independent replicates (*n* = 3).

Table S3. Demographic and clinical characteristics of the patients with periodontal disease.

Subject Code	Age	Race/Ethnicity	Gender	Diagnosis (patient-based)	Last Antibiotics	Last scaling and root planing or periodontal maintenance	ASA Type	BMI	Smoking	Medical condition	Medication
12-1	51	NA (decline to answer)	F	Periodontitis, stage II, localized, Grade B	>2 month	>2 month	I	24.7	None	None	None
12-2	61	NA (decline to answer)	F	Periodontitis, stage III generalized, Grade C	>2 month	>2 month	II	25.6	None	Diabetes; Hypothyroidism	Levothyroxine
12-3	41	NA (decline to answer)	F	Periodontitis, stage II, generalized, Grade B	>2 month	>2 month	I	21.3	None	None	None
12-4	42	NA (decline to answer)	F	Periodontitis, stage III, generalized, Grade B	>2 month	>2 month	I	35.6	None	None	None
12-5	61	NA (decline to answer)	M	Periodontitis, stage III, generalized, Grade B	>2 month	>2 month	II	30.8	None	Hepatitis C; Glaucoma	Dorzolamide/Timolol Latonoprost Tamsulosin HCl
12-6	36	NA (decline to answer)	M	Periodontitis, stage III, generalized, Grade B	>2 month	>2 month	I	20.2	None	None	None
12-7	65	NA (decline to answer)	M	Periodontitis, stage III, generalized, Grade B	>2 month	>2 month	II	26.4	None	Hypothyrodism	Levothyroxine Stelara

Table S4. Site-specific clinical parameters of GCF collection sites.

ID	Subject Code	Sample site	Probing Pocket Depth (mm)	Clinical Attachment loss (mm)	Bleeding on Probing
1	12-1	5DB	2	1	-
2	12-1	31MB	4	3	-
3	12-1	3DB	4	5	-
4	12-1	3MB	3	3	-
5	12-1	4DB	2	2	-
6	12-2	31DB	5	5	-
7	12-2	2MB	5	4	+
8	12-3	3DP	5	3	+
9	12-3	2MP	4	2	+
10	12-3	3MP	5	3	+
11	12-3	2MB	4	3	-
12	12-4	4DB	3	2	-
13	12-4	3MP	6	5	+
14	12-4	3MB	5	2	+
15	12-4	4DP	4	3	+
16	12-5	2DP	7	8	+
17	12-5	3MP	5	4	+
18	12-5	3DB	6	4	+
19	12-5	4DB	3	2	-
20	12-5	2DB	7	8	+
21	12-6	12MB	3	1	-
22	12-6	14MB	6	3	+
23	12-6	15DP	9	7	-
24	12-6	14MP	5	3	+
25	12-6	13MB	5	2	-
26	12-7	20MB	4	2	-
27	12-7	19MB	4	4	-
28	12-7	18MB	3	4	-

Correlation between absorbance variation and bacterial load

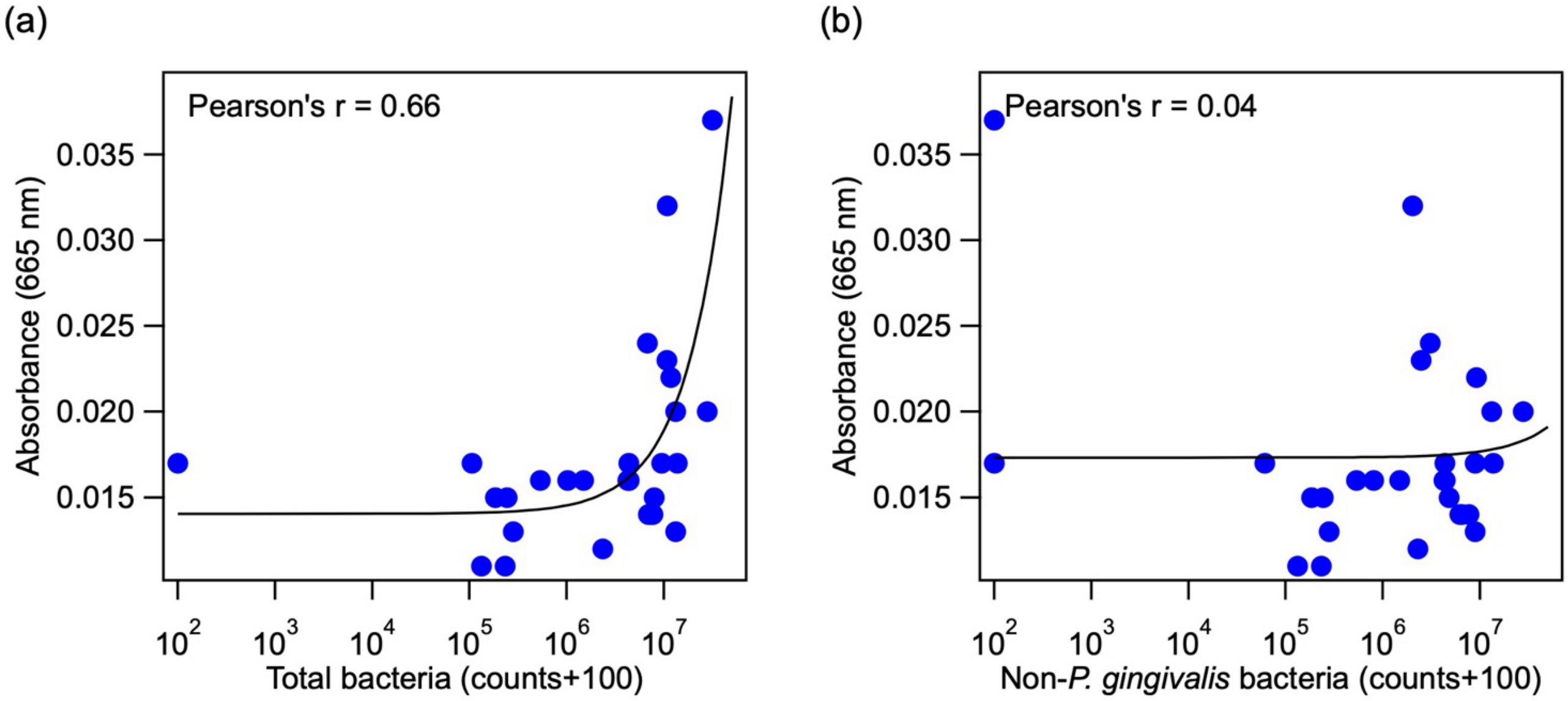

Figure S18. Correlation between the absorbance variation of the supernatant and the number of (a) total bacterial load and (b) non-*P. gingivalis* bacterial load detected by qPCR. For one sample, the *P. gingivalis* count exceeded the total bacterial count, resulting in a negative calculated value for the non-*P. gingivalis* bacterial load. This value was therefore set to 0 for the analysis. To include samples with zero counts, all values were shifted by adding 100, and the x-axis is shown as bacteria (counts + 100).
